# Co-cultivation of Mortierellaceae with ***Pseudomonas helmanticensis*** affects both their growth and volatilome

**DOI:** 10.1038/s41598-023-29134-6

**Published:** 2023-02-07

**Authors:** Maraike Probst, Anusha Telagathoti, Bianka Siewert, Iuliia Khomenko, Emanuela Betta, Franco Biasioli, Ursula Peintner

**Affiliations:** 1 Department of Microbiology, Universität Innsbruck, Technikerstrasse 25, 6020 Innsbruck, Austria; 2 Institute of Pharmacy, Center for Chemistry and Biomedicine, Center for Molecular Biosciences Innsbruck (CMBI), Universität Innsbruck, Innrain 80 - 82/IV, 6020 Innsbruck, Austria; 3 Research and Innovation Centre, Fondazione Edmund Mach, Via Edmund Mach 1, 38010 San Michele all’Adige, Italy

**Keywords:** Microbial ecology, Environmental microbiology, Fungi

## Abstract

Volatile organic compounds (VOCs) might mediate microbial interactions, especially in spatially structured environments, such as soil. However, the variety and specificity of VOC production are poorly understood. Here, we studied 25 Mortierellaceae strains belonging to the genera *Linnemannia* and *Entomortierella* in both pure and co-culture with *Pseudomonas helmanticensis* under laboratory conditions. We analysed both the fungal growth depending on co-cultivation and the cultures’ volatilomes applying proton-transfer-reaction time-of-flight and gas chromatography-mass spectrometry (PTR-ToF-MS and GC–MS). In a strain-specific manner, we found the fungi’s radial growth rate and colony morphology affected by the presence of *P. helmanticensis*. The fungus seemed to generally reduce the bacterial growth. The volatilomes of the fungal and bacterial pure and co-cultures were diverse. While the fungi frequently consumed VOCs, *P. helmanticensis* produced a higher diversity and amount of VOCs than any fungal strain. Our results support that both the pure and co-culture volatilomes are taxonomically conserved. Taken together, our data supports the relevance of VOCs in Mortierellaceae-*P. helmanticensis* interaction. We also discuss individual VOCs that appear relevant in the interaction.

## Introduction

Soil harbours the most diverse microbial communities known, thus it is a hot spot for interactions like sensing, collaboration, and defence^1^. Interactions among microorganisms are diverse, ranging from antagonistic to mutualistic, and potentially modulate the morphology or the metabolic pattern of both involved partners^2^. Positive interactions are often species-specific, and have a long co-evolutionary history, as reported for early emerging fungi and endobacteria^3^. Neutral or negative interactions are often unspecific and transitional. Selected soil bacteria use fungal hyphae as transport routes^4–6^, thus affecting fungal physiology^7^. Multiple interactions occur contemporaneously under natural conditions: a fungus can establish mutualistic interactions with one partner, but develop antagonistic ones with other soil organisms^8^. In addition to the partners involved, interactions depend on the environmental conditions: The type of interaction can also change with time or environmental conditions, e.g. nutrient availability, temperature, soil pH, and moisture content. This makes it challenging to study these interactions, especially in their natural habitat^9^.

Interactions among soil organisms can be mediated by volatile organic compounds (VOCs), as they have the ability to diffuse through both air and water pockets in the soil (low molecular weight, low boiling point). VOCs can also significantly affect the well-being and survival of associated plants and animals^10–12^. Microbial VOCs received a significant amount of attention during the last two decades, but their hosts and potential recipients as well as their ecologic role remain poorly understood^13^. This restricted knowledge is one factor limiting our understanding of mechanisms triggered by VOCs. A deeper, more systematic knowledge concerning species-specific VOC patterns is limited to a handful of fungi, e.g. *Trichoderma*^14^ and some Mortierellaceae species^15^. One recent landmark study highlighted the ecological importance of VOCs for the initiation and regulation of fungal interactions: based on 45 species of fungi, the authors showed that VOC patterns can describe both the phyla and trophic mode of fungi^16^.

Mortierellaceae is a family of soil fungi with a wide, global scale distribution^17^. Species belonging to this group are often associated to bacteria^18,19^. However, bacteria are not randomly associated to these fungi, as selected *Pseudomonas* species were unusually often detected in association with Mortierellaceae. *Pseudomonas* spp. are abundant and widespread in soil^20,21^. *Pseudomonas helmanticensis* was discovered in Spanish forest soils^22^, and it was frequently detected in association with Mortierellaceae species in many different habitat types of the alpine range^19^. However, it is unknown if these bacteria prefer a specific fungal host or how they affect it.

It is known that endobacteria associated to *Linnemannia elongata* (formerly *Mortierella elongata;* Mortierellaceae) caused changes in VOC production of their hosts^23^. This interesting finding raised the questions if Mortierellaceae VOC production is specific or conserved, and if the production of VOCs involved in fungal-bacterial interactions is specific or conserved. One of our previous studies addressed the first question^15^. Based on a large number of different Mortierellaceae species and strains, we showed that (i) Mortierellaceae produce a wide range of VOCs in pure cultures and that (ii) the volatilome is species-specific in composition and concentrations. We concluded that VOCs potentially provide advantages to the producing organisms in the competitive soil environment, and might therefore be ecologically meaningful. However, knowledge concerning potential changes in VOC production during interaction with other soil organisms is still lacking.

Hence, we cultivated five *Linnemannia* species and one *Entomortierella* species (outgroup) both in pure and co-culture with *P. helmanticensis* (3–6 strains each species). We observed the fungal growth behaviour and the (pure and co-)cultures’ volatilomes. Within this setup, we addressed the influence of *P. helmanticensis* co-cultivation on both, the fungal growth behaviour and the cultures’ VOC production. The growth behaviour was assessed based on daily radial growth rate and morphology. Volatilomes were analyzed in pure and co-culture using proton-transfer-reaction time-of-flight mass-spectrometry (PTR-ToF-MS) for a complete screening and gas chromatography mass-spectrometry (GC–MS) on a sample subset to support compound identification.

We hypothesized: (i) a taxonomically conserved growth behaviour and volatilome among the fungal species and between the fungal genera *Linnemannia* and *Entomortierella* in both presence and absence of *P. helmanticensis*; (ii) the co-cultivation and strain heterogeneity to strongly affect both growth and volatilome; (iii) the growth behaviour to be correlated with the volatilome. Furthermore, we aimed at identifying individual VOCs that might be regulated in Mortierellaceae-*P. helmanticensis* interaction; those can be tested in future lab experiments.

## Materials and methods

### Cultivation and isolation of *Linnemannia* and* Entomortierella* strains

All the *Linnemannia* and *Entomortierella* strains used in this study were isolated from soil samples originating from different habitats (sub-alpine, alpine forest soils, snowfields, and barren ground from glacier successional sites) in Austria (SI Table S1). Frequently, we found *Pseudomonas helmanticensis* (OM333167) associated to the fungal isolates^15^. Most of the *Linnemannia* and *Entomortierella* strains studied here were isolated by direct plating as described in our previous study^19^. The *Linnemannia* and *Entomortierella* isolates used here were carefully selected based on the results of the phylogenetic analysis of rDNA-ITS sequences (SI Fig. S1). Overall, five *Linnemannia* species were selected; an *Entomortierella* species was used as an outgroup. For each clade representing a species, three to five strains were used as biological replicates.

**Figure 1:**
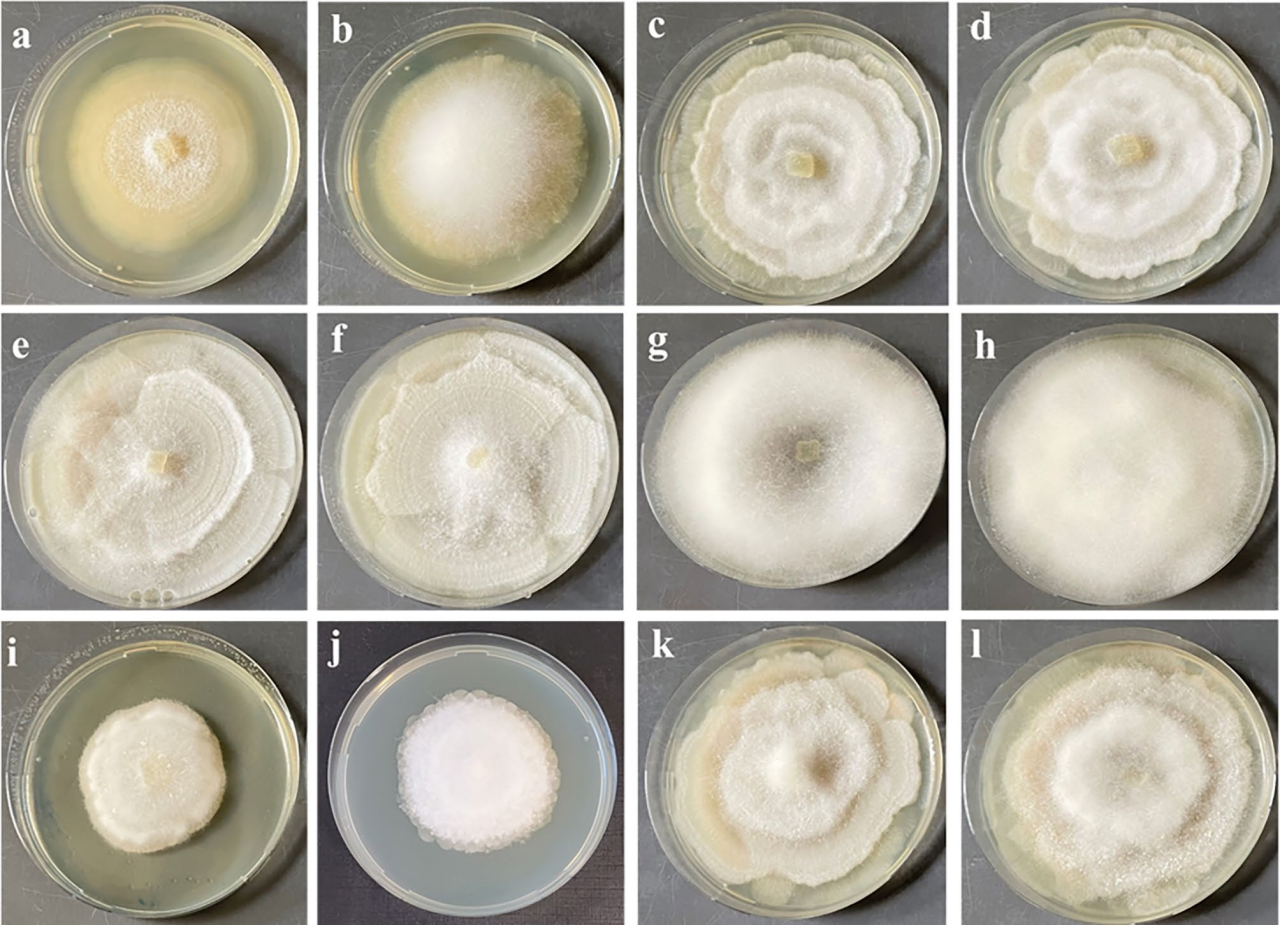
Morphology of *Linnemannia* and *Entomortierella* species on PDA in pure culture (second and forth column) and in co-culture with *Pseudomonas helmanticensis* (first and third column) incubated at 10 °C for ten days. One exemplary strain was randomly selected for each species. (**a**,**b**) *E. galaxiae,* (**c**,**d**) *L. sclerotiella,* (**e**,**f**) *L. gamsii,* (**g**,**h**) *L. hyalina,* (**i**,**j**) *L. solitaria,* (**k**,**l**) *L. exigua*. The formation of aerial mycelium is often reduced in co-cultivated strains, and the typical rosette-like colony morphology is less pronounced.

In order to obtain pure fungal cultures without any bacterial association, the selected strains were grown on potato dextrose agar (PDA-26.5 g of potato dextrose and 15 g of agar in 1 L of deionized water, Carl Roth GmbH, Karlsruhe, Germany) with the sterile addition of antibiotics: streptomycin sulfate (final concentration 0.1 g L^−1^ medium) was dissolved in water; tetracycline (final concentration 0.05 g L^−1^ medium) was dissolved in ethanol. For curation, the PDA plates were incubated at 10 °C.

To obtain a pure culture of *Pseudomonas helmanticensis* (OM333167), the fungal mycelium was crushed using a pestle, suspended in 100 µl of water, and centrifuged at maximum speed. The supernatant was then plated on Tryptic Soy Agar (TSA) and incubated at 25 °C overnight. A colony was picked carefully from the obtained colonies and re-streaked for further experiments. To confirm the identity of the bacterial culture used for the co-plating experiment, its 16S region was amplified using primers 8F/1492R^23^ (AGAGTTTGATCCTGGCTCAG, GGTTACCTTGTTACGACTT), the PCR products were unidirectionally sequenced by Microsynth AG (Balgach, Switzerland) using the Sanger method. Sequence identity was confirmed by the NCBI blast^24^ limited to type sequences using 99% sequence similarity and 99% query cover.

### Co-cultivation of *Linnemannia* and *Entomortierella* with *Pseudomonas helmanticensis*

Fungal pure cultures of *Linnemannia* and *Entomortierella*, respectively, were co-cultivated with *P. helmanticensis* on PDA media. The plates were incubated at 10 °C for 5 d until the fungal mycelium was completely covering the bacterial colonies. Fungal aerial mycelium was then inoculated on PDA without antibiotics and incubated at 10 °C for 7 d. After the incubation period, the fungal mycelium was screened for the associated bacterium by PCR amplification of the 16S region. These successfully co-cultivated fungal-*P*. *helmanticensis* plates were used for further experiments.

### Fungal growth

To understand the influence of *P. helmanticensis*’ presence on the fungal cultures in terms of fungal radial growth rate per day, two different media were used, nutrient rich PDA and nutrient poor LcA^25^. On each medium, both the pure and co-cultivated *Linnemannia* and *Entomortierella* isolates were plated in five replicates and incubated at 10 °C. The colony diameter was measured daily until the colonies reached their maximum. From the daily diameter, the average gain per day was calculated as daily radial growth rate. The growth rate covers the duration of the experiment and can be expressed as fungal daily radial growth rates. However, it can account only for the two-dimensional horizontal spread of the culture; differences in aerial mycelium production (growth into the dimension height) are not considered. Here, the growth rate of the co-cultures is the fungal growth rate in co-culture, as the bacterial growth cannot be visually quantified.

### Cultivation of *Linnemannia* and *Entomortierella* for volatilome analysis

The measurement of VOCs was carried out in 20 mL glass vials containing 5 mL of PDA slant agar medium and closed with a screw cap with a silicon/PTFE septum. An agar block of approximately 3 × 3 × 3 mm containing mycelium from pure and co-cultivated isolates was used as an inoculum for each vial. For each strain, three technical replicates were plated. Inoculated vials were incubated at 16 °C with a loosely attached screw to ensure air supply for three days in darkness. Pure bacterial cultures were measured in five technical replicates using the same conditions. For controls, vials containing medium only (without fungal/bacterium inoculum) and empty vials were used. They were incubated under the same conditions and included in the volatilome analysis.

### VOC measurement

The VOC measurement was carried using both proton transfer reaction time of flight–mass-spectrometry (PTR-ToF-MS) and gas chromatography mass spectrometry (GC–MS). The PTR-ToF-MS was used for sensitive and diverse volatilome profiling; GC–MS was used to identify individual compounds detected in PTR-ToF-MS.

Using PTR-ToF-MS the headspace of the vials was analysed over a period of 4 d (day 4–day 7). During the measurement, every sample was measured four times, resulting in four (multivariate) volatilome data points for each sample. The duration between time points was 1 d and similar for all samples. Moreover, every sample was analysed in three technical replicates, i.e. every strain was inoculated into three different vials (3 × pure culture, 3 × co-culture). For GC–MS, one measurement was performed directly after the last PTR-ToF-MS measurement for one randomly picked technical replicate. So, each fungal strain both in pure and co-culture as well as the bacterial pure culture were analysed once in GC–MS.

PTR-ToF-MS measurements were performed on a commercial PTR-ToF-MS 8000 apparatus (Ionicon Analytik GmbH, Innsbruck, Austria). In order to ensure similar time intervals between measurements and across sample groups, an adapted autosampler (MPS Multipurpose Sampler, GERSTEL, Mühlheim an der Ruhr, Germany) was connected. Following the three technical replicates, three identical sets of sample groups were prepared and they were randomly placed into three holders for the autosampler. Measurements were performed on the headspace of each vial by direct injection into the instrument.

The drift tube conditions were as follows: 2.8 mbar drift pressure, 110 °C drift tube temperature, 530 V drift voltage. The ion funnel was on and it was operated at the end of the drift tube in order to improve sensitivity^26^, thereby leading to an E/N ratio of about 140 Townsend (Td) (E = strength of the electric field, N = gas number density (1 Td = 10^−17^ V cm^2^)). Per channel of ToF acquisition, the sampling time was 0.1 ns, which results a total number of 350,000 channels for a mass spectrum ranging up to m/z = 400. A PTR-MS inlet collected the sample headspace at a flow of 40 sccm (standard cubic centimeters per minute) for 60 cycles (total analysis time of 60 s/sample). The vials were continuously flushed with air to prevent pressure drop and allow for sufficient oxygen supply for fungal growth.

The data were processed as described by Capellin et al.^27^ There were 384 mass peaks in the raw dataset. The dataset was filtered by noise and by applying correlation coefficient thresholds. Peaks with no significant differences from the blank samples were removed; highly autocorrelated peaks were removed (|r|> 0.99). For the most part, the latter correspond to isotopologues of monoisotopic masses^28^.

The concentrations in ppbV (part per billion by volume) of tentatively identified compounds were calculated according to Lindinger et al.^29^ A constant value of 2.0 × 10^−9^ cm^3^ s^−1^ was used for the reaction coefficient to estimate absolute concentrations.

HS-SPME/GC–MS (Head space Solid Phase Micro Extraction coupled with Gas chromatography Mass Spectrometry) measurements were performed with an adaptation of a previously described method^30^. Vials were temporarily moved from the PTR-MS autosampler on the autosampler of the GC (CTC combiPAL, CTC Analytics AG, Zwingen, Switzerland) at the same temperature and equilibrated for 10 min. Solid-phase microextraction fiber (DVB/CAR/PDMS, Supelco, Bellefonte, PA, USA) was exposed for 30 min in the vial headspace. The fiber was then desorbed at 250 °C in the injector port of a GC interfaced with a mass detector operating in electron ionization (EI) mode (70 eV) with a scan range of m/z 33–350 (GC–MS Clarus500, PerkinElmer, Norwalk CT, USA). Separation was carried out in an HP-INNOWax fused silica capillary column (30 m, 0.32-mm ID, 0.5-µm film thickness; Agilent Technologies, Palo Alto, CA, USA). The GC oven operated for 4 min at 40 °C, then increased at 5 °C min^−1^ until 250 °C, which was maintained for 2 min. Helium was used as carrier gas at a constant flow of 2 mL min^−1^. Compound identification was based on mass spectra matching with the standard NIST/EPA/NIH (NIST 14) and Wiley 7th Mass Spectral Libraries, and linear retention indices (LRI) compared with the literature. LRI were calculated under the same chromatographic conditions after injection of a C7–C30 n-alkane series (Supelco, Bellefonte, PA, USA).

The identity of the tentatively annotated PTR-ToF-MS mass peaks was confirmed by correlation (both Spearman and Pearson) of the PTR-ToF-MS mass peak concentrations to their counterpart concentrations measured by GC–MS. The PTR-ToF-MS mass peaks and the GC–MS compounds were matched based on their tentative annotation and identification, respectively. (For example, the concentrations of mass peak ms48.0158 tentatively annotated as ethanol were correlated to the GC–MS compound identified as ethanol.) As for GC–MS one replicate was measured, correlation was performed using the mean mass peak concentration obtained by PTR-ToF-MS across all time points measured. For significant correlations, mass peak annotations were accepted; for insignificant correlations, mass peak annotations were indicated by the phrasing ‘tentatively annotated as’.

### Statistical analysis

All statistical analyses were performed in R v4.0.2^31^. Unless stated otherwise, a confidence interval of 95% was applied (alpha = 0.05) for all tests. Scripts can be found on Github: Maraikep/Mortierellaceae_VOCs.

Fungal daily radial growth rate was analysed using analysis of variance (ANOVA). An overall model was calculated including the experimental factors medium (PDA and LcA), species, strain and cultivation mode (pure and co-cultures). The factor strain was nested within species. Interaction effects of experimental factors were considered. The model was reduced to include only significant independent variables. Residuals were normally and variances were homogeneously distributed as confirmed by residual diagnostics.

From the PTR-ToF-MS raw data, we first checked the number and concentrations of VOCs in both PDA medium and samples. As PTR-ToF-MS is a very sensitive machine able to detect very small quantities, almost all VOCs were detected in the PDA. In order to ensure that the VOC emission of the PDA did not mask the volatilome patterns among sample groups and between pure and co-cultures, we set different thresholds as detection limit. The thresholds (0, 0.05, 0.2, 0.5, 1, 5, 10, 20 ppbV) were selected based on the distribution of VOC concentrations detected in PDA (SI Fig. S2). Across thresholds we observed both consumption and production of VOCs for all sample groups. In other words, we observed that some VOCs with high concentrations in PDA were lower in concentration in the cultures (consumption). Similarly, across thresholds we observed higher VOC concentrations in the cultures compared to PDA and we observed VOCs detected only in the cultures and not in PDA (production). The probability of VOC consumption was modelled using generalized linear models. The binomial distribution and logit function were assumed as theoretical distribution and link function, respectively. Models were calculated for each threshold and then compared.

For further analysis, VOC concentrations were corrected for PDA emission. We did not set a threshold for this correction, in order to have maximum variance for modelling: Three technical replications of vials containing only PDA medium were included. They were randomly distributed across the sample set and measured in all time points, similar to the controls. For the majority of mass peaks, their concentrations were both stable across time and comparable among the PDA controls as tested using graphs and linear modelling, respectively. We used the PDA control concentrations measured and tested if each individual sample measurement differed significantly from this PDA null distribution (12 measurement points). If the T-test indicated significant differences among sample concentration and PDA controls, the difference in concentrations (mean PDA and sample) was used as true sample value. The resulting positive concentrations for the samples indicate production of the VOC above PDA. The resulting negative concentrations for the samples indicate that the VOC was emitted by PDA and consumed by the culture. If the difference was insignificant, the concentration measured resulted from the PDA medium and was set to zero for the individual sample. For those few mass peaks for which the linear model using time as independent variable returned significant, we used the model predicted concentration and standard deviation for creating a normal distribution. From this normal distribution, we randomly picked 12 values and used them as PDA null distribution as before. This normalized dataset was used for all further analysis.

In order to test the predictability of the experimental factors time, species, strain, and cultivation mode, we applied linear discriminant analysis (LDA) on scaled data. For both, species and strain their respective interaction with cultivation mode was predicted. For all experimental factors, including the interaction effects, but except cultivation mode, homogeneity of variances was confirmed graphically using covEllipses from R package heplots^32^. Within the factor cultivation mode, volatilomes of the co-cultures had a higher variability compared to pure cultures. With very few exceptions, variables were normally distributed as tested by Shapiro test. Each LDA was trained on a 70% random subset of the overall dataset. The accuracy of the LDA was tested on those 30% of the data that were not used for training the model; it was interpreted as the predictability of (interaction of) experimental factor(s). Both the test and training set represented all sample groups. The association of the individual volatiles (= volatile compounds represented by mass peaks) with the LDs (Linear Discriminants) was interpreted; only LDs with a minimum of 10% variance were considered. The association scores of the volatiles were normally distributed and those scores in the extreme ends of the distribution were considered volatiles with high association to the respective LDs, i.e. volatiles associated to the sample grouping along LDs.

General linear modelling was used (i) to predict the bacterial contribution to the volatilome of each co-culture of fungal strain and *P. helmanticensis*, and (ii) to identify volatiles that might be affected by cultivation mode. In other words, we assumed (i) that the majority of volatiles composing the volatilomes of both the bacterial and fungal pure cultures are linearly dependent on cell density and (ii) that in co-culture the majority of volatiles behave in an additive manner. Based on these assumptions, for each strain separately, we calculated three models. First, we used all VOCs detected and predicted both the fungal and the bacterial contribution of the co-culture volatilome from both pure culture volatilomes as follows:

1 $$Voc\left(co-culture\right)=a*Voc\left(P.helmanticensis\right)+b*Voc\left(fungalstrain\right)$$

Second, we used only those VOCs produced by *P. helmanticensis* and neither consumed nor produced by the respective fungal strain. Then, we predicted the concentrations of those VOCs in co-culture from the bacterial pure culture, thereby estimating only the bacterial coefficient, as follows:

2 $$Voc\left(co-culture\right)=a*Voc\left(P.helmanticensis\right)$$

Third, we used the coefficient of model 2 to predict the bacterial production of each VOC in co-culture. Then, we subtracted the predicted concentration from the measured VOC concentration in co-culture in order to obtain the predicted fungal contribution to the co-culture volatilome. This predicted fungal volatilome in co-culture we used for estimating only the fungal coefficient:

3 $$Voc\left({predictedco-culture}_{fungalstrain}\right)=a*Voc\left(fungalstrain\right)$$

The coefficients, a and b, of the Eqs. (1–3) can be considered as a proxy for the bacterial and fungal contribution to the co-culture volatilome, i.e. the extrapolated bacterial and fungal growth, respectively. They are independent from each other and can be compared across models. The model assumptions were checked using residual diagnostics. The residuals of the model were homogeneously distributed. Normal distribution can also be assumed: the differences of mean concentrations across a number of replicates were used for modelling (central limit theorem). It should be noted that only those residuals that behave in an additive manner can be expected to follow normal distribution. Those that obviously did not follow normal distribution were, therefore, not necessarily indicative for a bad model fit, but potentially ecologically relevant.

As a proof of concept, we correlated (Pearson and Spearman) the coefficients estimated in the linear volatilome models to (i) the differences in fungal daily radial growth rate, i.e., the mean differences between fungal pure cultures and co-cultures and (ii) the Euclidean centroid distances (along all model LDs) between the LD scores of the fungal pure cultures and co-cultures. For growth behaviour, we tested two different scenarios, which did not differ in their interpretation: non-significant differences in fungal daily radial growth rate among pure fungal cultures and co-cultures were set to a mean difference of zero and raw mean differences among the two groups were correlated to the coefficients.

## Results

### The growth behaviour of *Linnemannia* is strain-specific

Most strains showed comparable morphological characteristics on both media as well as in pure and co-culture. However, *Linnemannia solitaria* and *Entomortierella galaxiae* produced more aerial mycelium on PDA compared to LcA. There was more/less aerial mycelium in co-cultures with *P. helmanticensis* compared to pure cultures depending on the strain (Fig. 1, SI Fig. S3).

**Figure 2:**
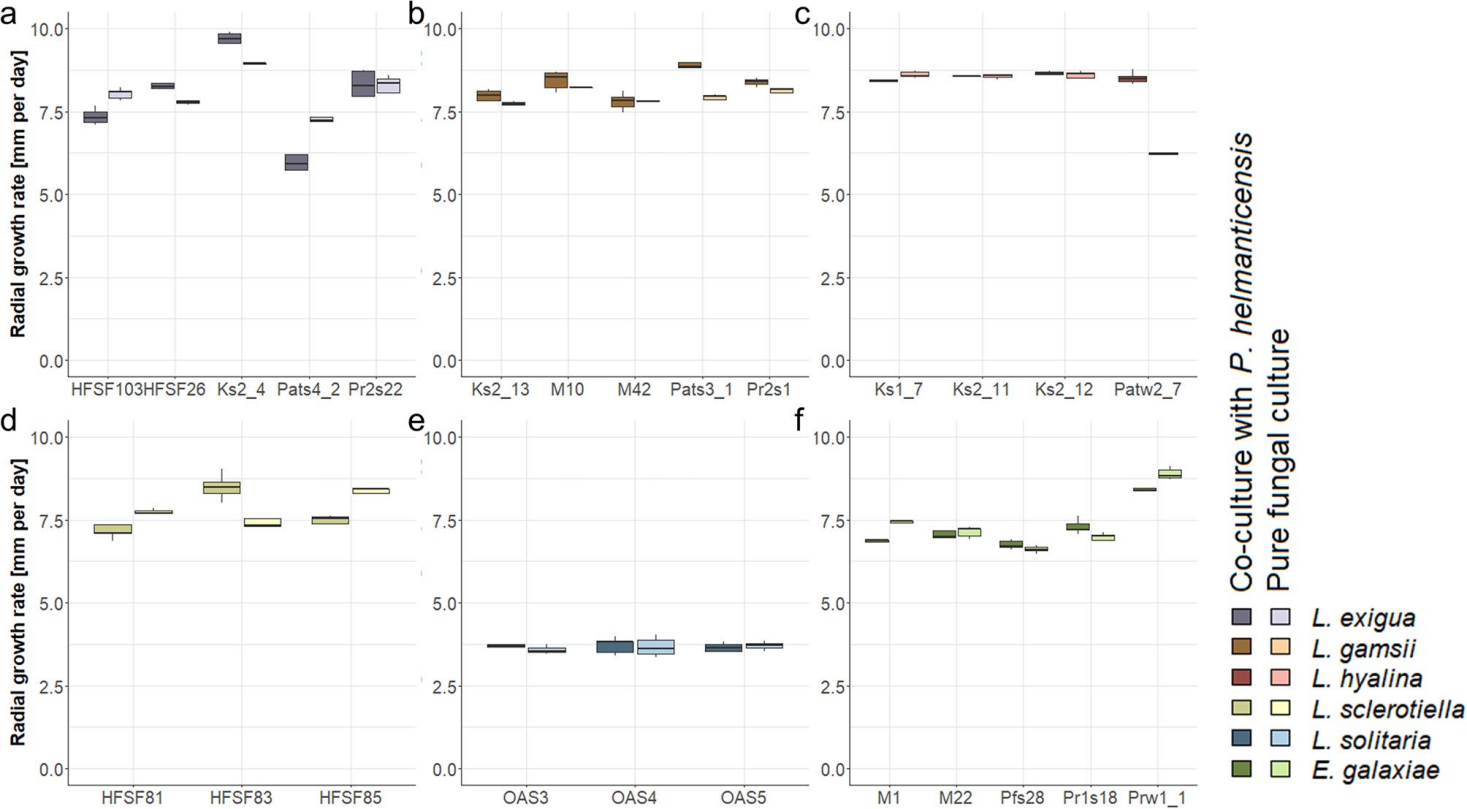
Daily radial growth rate of pure *Linnemannia* and *Entomortierella* cultures as well as co-cultures with *P. helmanticensis* on nutrient rich PDA medium*.* (**a**) *L. exigua,* (**b**) *L. gamsii,* (**c**) *L. hyalina,* (**d**) *L. sclerotiella,* (**e**) *L. solitaria,* (**f**) *E. galaxiae.*

The comparison of *Linnemannia* and *E. galaxiae* daily radial growth rates did not support a difference between these genera (*p* ≥ 0.3). The overall linear model indicated that the fungal daily growth rates mainly differed among species (Table 1). In addition, the effect of strains highlighted the heterogeneity among strains within species (Fig. 2, SI Figs. S4, S5). Although there was no relevant main effect of medium on the daily radial growth rate of the fungi, the medium did affect the fungi in a strain-specific manner (Table 1, Fig. 2, SI Figs. S4, S5). On nutrient poor LcA, the fungal daily radial growth rates were reduced for all species, except for *L. solitaria*, which grew better on LcA (SI Figs. S3, S4).

**Table 1:** The effect of experimental factors on the fungal daily radial growth rate.

Variables	Df	Sum Sq	Mean Sq	F value	Pr(> F)	Effect
Species	5	409.7	81.9	851.600	<< 0.001	49.6
Media	1	1.0	1.0	10.470	0.00131	0.1
Co-Culture	1	5.4	5.4	55.650	<< 0.001	0.7
Strain in species	19	143.4	7.6	78.460	<< 0.001	17.4
Species:Media	5	145.2	29.1	301.890	<< 0.001	17.6
Species:Co-culture	5	6.2	1.3	12.950	<< 0.001	0.8
Strain in Species:Media	19	20.2	1.1	11.070	<< 0.001	2.4
Strain in Species:Co-culture	19	44.7	2.4	24.450	<< 0.001	5.4
Residuals	425	50.4	0.1			

F(74, 425) = 109; *p* << 0.001; adjusted R^2^ = 0.941. Effect represents the size of each effect calculated from the percentage of the sum of squares explained by the respective factor.

The main effect of co-plating *P. helmanticensis* on radial growth rate was small, yet significant (0.7%, *p* << 0.001), and highly strain specific (Fig. 2, SI Table S2). Given that the growth rate refers to only the fungus, those strains with differences in growth rates between pure cultures and co-cultures can be considered affected by co-cultivation. On PDA, six strains grew faster (mean ± standard deviation = 1.1 ± 0.69 mm d^−1^) and six strains grew slower (0.5 ± 0.21 mm d^−1^) when co-cultivated with the bacterium. On LDA, four strains grew faster (0.7 ± 0.42 mm d^−1^) and ten strains grew slower (0.9 ± 0.73 mm d^−1^) when co-cultivated with the bacterium.

**Table 2:** Linear models predicting the volatilome contribution of *Pseudomonas helmanticensis* and Mortierellaceae strains in co-cultures.

Species	Strain	Growth rate pure|co [mm d^-1^]	Model 1			Model 2			Model 3		
			Fungal coefficient	Bacterial coefficient	adj.R^2^	Bacterial coefficient	adj.R^2^	#VOCs	Fungal coefficient	adj.R^2^	#VOCs
*L. exigua*	HFSF103	8.1 > 7.3*	1.8 ± 0.16**	0.20 ± 0.023**	0.739	0.29 ± 0.029**	0.797	27	1.4 ± 0.30**	0.205	34
*L. exigua*	HFSF26	ns	0.7 ± 0.20**	0.29 ± 0.019**	0.723	0.23 ± 0.039**	0.539	30	1.01 ± 0.052**	0.292	33
*L. exigua*	Ks2_4	8.9 < 9.8*	0.3 ± 0.17	0.29 ± 0.023**	0.699	0.14 ± 0.028**	0.472	28	0.8 ± 0.28*	0.287	39
*L. exigua*	Pats4_2	ns	1.1 ± 0.18**	0.27 ± 0.024**	0.757	0.33 ± 0.011**	0.981	18	0.9 ± 0.11**	0.271	55
*L. exigua*	Pr2s22	ns	0.66 ± 0.077**	0.059 ± 0.0086**	0.633	0.08 ± 0.019**	0.365	31	0.55 ± 0.06**	0.06	37
*L. gamsii*	Ks2_13	7.7 < 8.0*	0.25 ± 0.094*	0.20 ± 0.014**	0.75	0.11 ± 0.023**	0.447	25	0.50 ± 0.030**	0.204	30
*L. gamsii*	M10	ns	0.19 ± 0.108	0.20 ± 0.014**	0.71	0.16 ± 0.023**	0.647	29	0.51 ± 0.039**	0.202	27
*L. gamsii*	M42	ns	0.02 ± 0.133	0.27 ± 0.015**	0.769	0.27 ± 0.024**	0.833	26	− 0.03 ± 0.087	0.273	29
*L. gamsii*	Pats3_1	7.9 < 9.0*	0.3 ± 0.16*	0.22 ± 0.018**	0.664	0.28 ± 0.047**	0.524	31	− 0.17 ± 0.134	0.219	26
*L. gamsii*	Pr2s1	ns	0.34 ± 0.066**	0.18 ± 0.016**	0.688	0.14 ± 0.031**	0.45	26	0.21 ± 0.017**	0.179	35
*L. hyalina*	Ks1_7	8.6 > 8.4*	0.45 ± 0.098**	0.18 ± 0.015**	0.679	0.37 ± 0.043**	0.692	33	0.09 ± 0.086	0.182	31
*L. hyalina*	Ks2_11	ns	0.59 ± 0.086**	0.17 ± 0.014**	0.699	0.21 ± 0.033**	0.561	32	0.63 ± 0.068**	0.173	29
*L. hyalina*	Ks2_12	ns	0.6 ± 0.11**	0.24 ± 0.015**	0.762	0.29 ± 0.047**	0.52	35	0.36 ± 0.041**	0.237	28
*L. hyalina*	Patw2_7	6.3 < 8.5*	0.14 ± 0.035**	0.22 ± 0.012**	0.781	0.13 ± 0.019**	0.63	30	0.3 ± 0.11*	0.224	29
*L. sclerotiella*	HFSF81	7.7 > 7.2*	0.89 ± 0.036**	0.01 ± 0.036	0.877	− 0.01 ± 0.011	− 0.054	10	0.91 ± 0.020**	0.012	80
*L. sclerotiella*	HFSF83	7.4 < 8.5*	0.2 ± 0.14	0.31 ± 0.019**	0.771	0.4 ± 0.059**	0.639	26	− 0.13 ± 0.097	0.312	40
*L. sclerotiella*	HFSF85	8.3 > 7.5*	0.5 ± 0.13**	0.27 ± 0.019**	0.751	0.2 ± 0.058**	0.457	25	0.46 ± 0.048**	0.272	41
*L. solitaria*	OAS3	ns	0.96 ± 0.037**	0.022 ± 0.0042**	0.911	0.03 ± 0.021	0.045	29	0.86 ± 0.030**	0.022	38
*L. solitaria*	OAS4	ns	0.93 ± 0.076**	0.040 ± 0.0089**	0.727	0.08 ± 0.028*	0.217	30	0.11 ± 0.060	0.038	37
*L. solitaria*	OAS5	ns	0.4 ± 0.22**	0.22 ± 0.022**	0.583	0.23 ± 0.055**	0.33	35	0.2 ± 0.11	0.218	28
*E. galaxiae*	M1	7.4 > 6.9*	− 0.2 ± 0.29	0.55 ± 0.035**	0.718	0.59 ± 0.082**	0.668	26	− 0.17 ± 0.138	0.552	29
*E. galaxiae*	M22	ns	− 0.1 ± 0.22	0.60 ± 0.025**	0.855	0.56 ± 0.042**	0.885	24	− 0.9 ± 0.11**	0.598	30
*E. galaxiae*	Pfs28	6.6 < 6.8*	1.00 ± 0.024**	0.01 ± 0.003	0.95	− 0.01 ± 0.002**	0.388	33	1.18 ± 0.035***	0.007	29
*E. galaxiae*	Pr1s18	6.9 < 7.3*	0.6 ± 0.30	0.57 ± 0.035**	0.787	0.53 ± 0.092**	0.539	29	0.6 ± 0.16***	0.572	40
*E. galaxiae*	Prw1_1	8.9 > 8.4*	− 0.01 ± 0.186	0.61 ± 0.029**	0.833	0.63 ± 0.073**	0.718	30	− 0.01 ± 0.188	0.609	28

Three models were calculated: (1) Co-culture volatilome predicted from both, fungal and bacterial pure cultures. All volatile organic compounds (VOCs) detected were used for prediction. (2) Co-culture predicted from pure *P. helmanticensis* cultures. The model considers only VOCs never produced/consumed in the respective fungal pure culture, but produced in *P. helmanticensis* pure cultures. (3) Bacterial contribution to the volatilome was predicted from model (1) and subtracted from the measured concentrations in the co-cultures. From this predicted volatilome, the fungal contribution to the co-culture volatilome was modelled. In none of the three models calculated, the intercept differed from zero. The coefficient of the equation can be considered as a proxy for the bacterial and fungal contribution to the co-culture volatilome, respectively, and indirectly for growth. Coefficient ± standard error. #VOCs = number of VOCs considered in the model. Significances: ns = not significant; * = *p* < 0.05; ** = *p* < 0.001.

Although the geographical origin location of a strain may affect the growth of a strain, here there was no clear pattern. Nevertheless, not all species were isolated from all locations, thus strain heterogeneity might have masked a potential location effect. With regards to historic or co-evolutionary effects of bacterial association, those strains isolated with a bacterium associated were not affected more frequently by co-cultivation (SI Tables S2, S3). Their growth was also not more likely to be in- or decreased by co-cultivation. Similarly, the re-cultivation with *P. helmanticensis* did not affect the daily radial growth rate of those fungal strains originally isolated in association with this bacterium in a similar manner, i.e. the same direction (de-/increase).

### The volatilome

**Figure 3:**
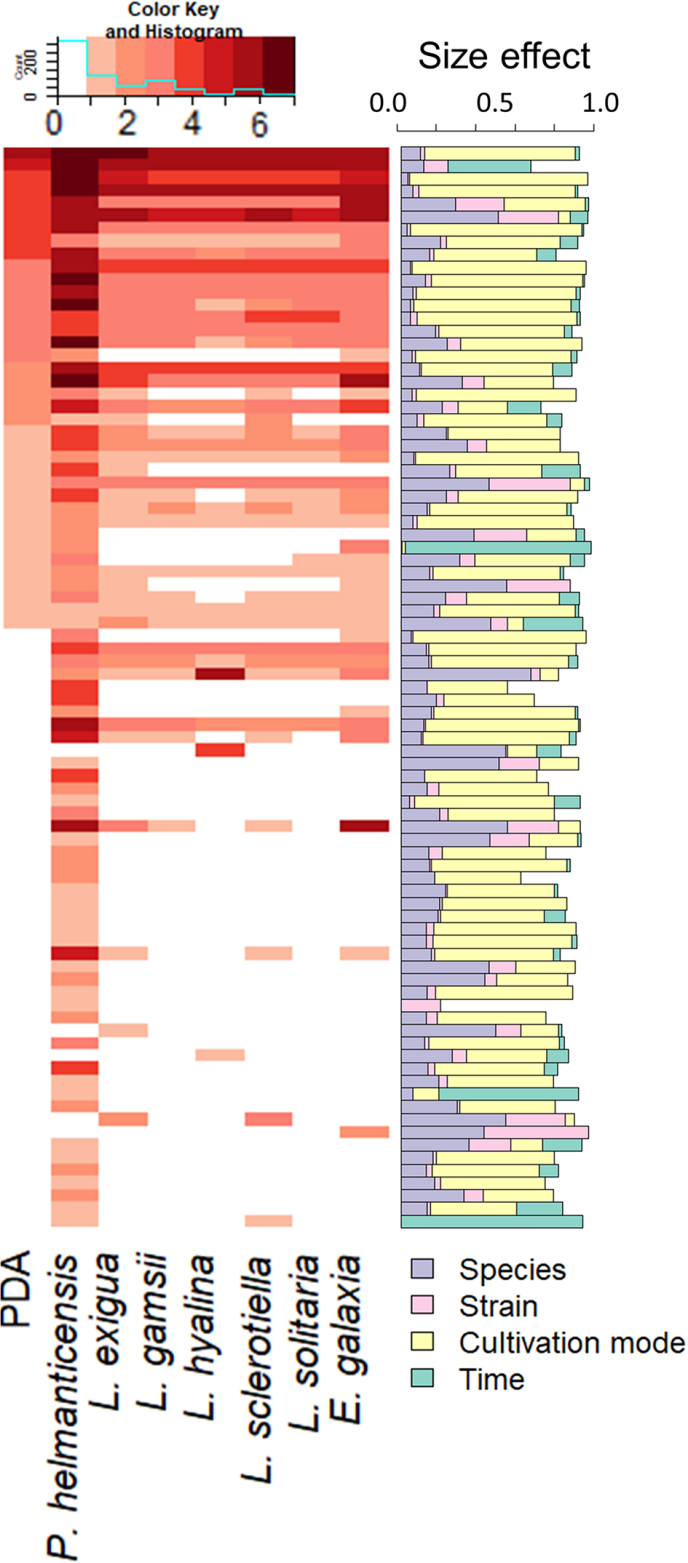
Concentrations of volatile organic compounds (VOCs) measured for sterile PDA medium and for pure cultures of *P. helmanticensis* and the *Linnemannia* and *Entomortierella* species studied here, and how they are affected by experimental factors (species, strain, cultivation mode and time). The concentrations of VOCs were measured by PTR-ToF-MS. The heatmap illustrates the mean concentrations of VOCs (rows) in PDA medium, *P. helmanticensis* pure cultures, and pure cultures of the fungal species (columns). For visualisation purposes, the concentrations were grouped: 0 = concentration < 0.2 bbpV; 1 = 0.2 ppbV ≤ concentration < 0.5 ppbV; 2 = 0.5 ppbV ≤ concentration < 1 ppbV; 3 = 1 ppbV ≤ concentration < 5 ppbV; 4 = 5 ppbV ≤ concentration < 10 ppbV; 5 = 10 ppbV ≤ concentration < 20 ppbV; 6 = 20 ppbV ≤ concentration < 50 ppbV; 7 = concentration ≥ 50 ppbV. The barchart visualizes the effect size of the experimental factors on the respective VOC concentration as calculated by ANOVA. The proportion of sum of squares explained by each factor was used as effect size.

**Figure 4:**
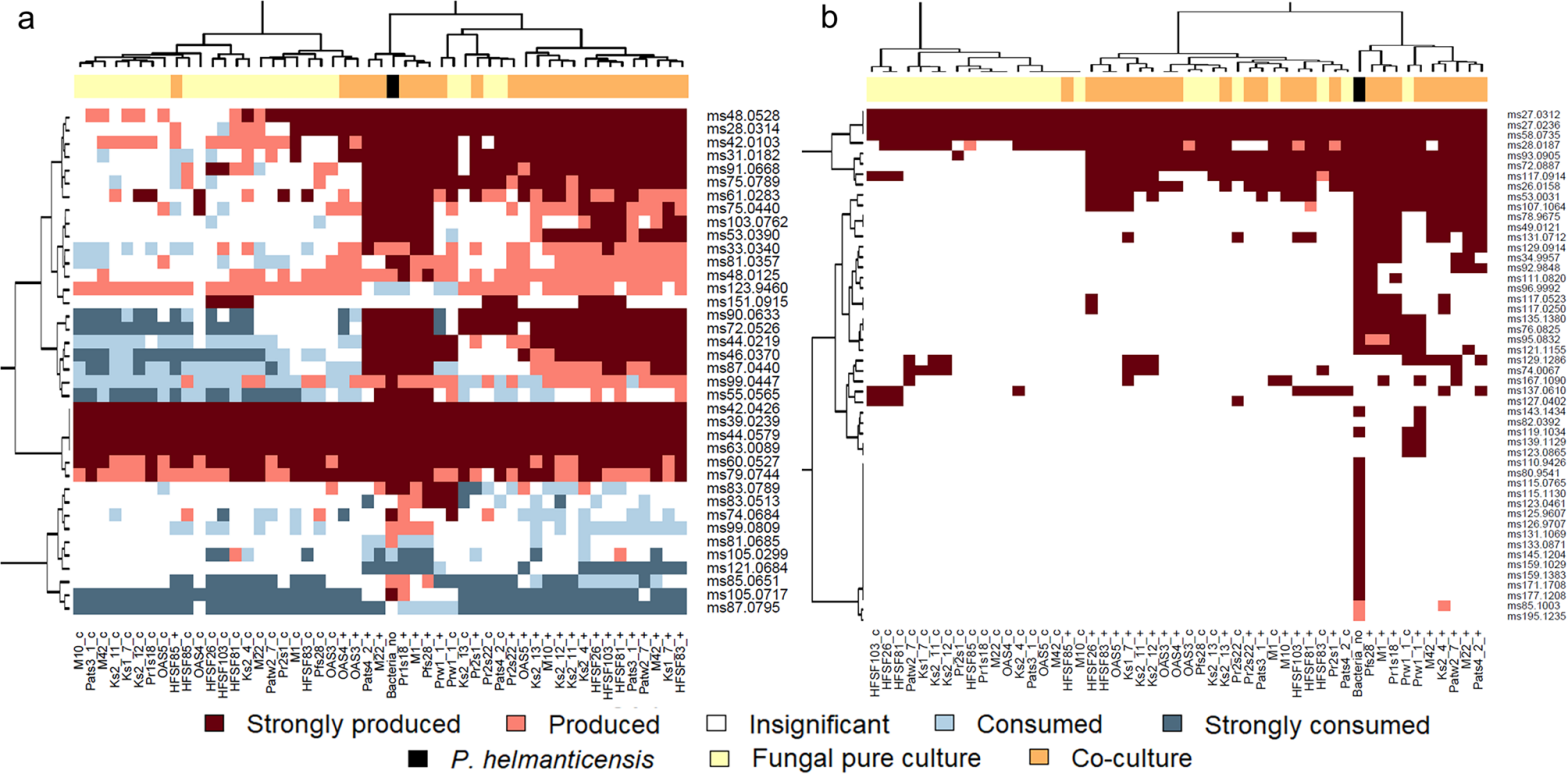
The production/consumption of volatile organic compounds (VOCs; rows) by pure fungal strains and pure *Pseudomonas helmanticensis,* as well as by their respective co-cultures (columns). A VOC detection limit of 0.2 ppbV was set. Both heatmaps visualise the mean VOC concentrations of the sample groups relative to the VOC concentration measured in PDA. (**a**) VOCs produced by PDA medium. The concentration in the respective sample group can be comparable to PDA, higher than PDA (production) or lower than PDA (consumption). (**b**) VOCs undetected in PDA medium. These VOCs were produced exclusively by the cultures. Production/Consumption of all VOCs indicated by colour was significant compared to PDA medium (*p* < 0.05). Strong production/consumption means that the respective VOC’s concentration was more than twofold higher/lower compared to its concentration measured for PDA medium.

We used two approaches to investigate the volatilome: On the one hand, we explored the effect of species, strains, cultivation mode (pure and co-culture), and time (Fig. 3) on the VOCs concentrations. As expected, the concentrations varied depending on all the experimental factors, mainly cultivation mode, but also species, strain, and partially on time. On the other hand, we asked which VOCs were produced/consumed by the fungus, *P. helmanticensis* and their co-cultures, respectively (Figs. 3, 4). All cultures both produced and consumed VOCs compared to the sterile PDA medium.

#### Stability over time

Over a period of four days, the volatilome of every sample (pure and co-culture) was measured four times using PTR-ToF-MS with similar time intervals between measurements and among samples. The rationale for this design was that we expected the volatilomes to be highly variable over time. Generally, timely changes were insignificant (50/113 VOCs) (Fig. 3). For those (63) VOCs that differed over time, with few exceptions, the time explained only a low percentage of variance (median = 5.4% variance, SI Fig. S6).

#### Fungal consumers and bacterial producers

Both the fungal and bacterial pure cultures as well as the PDA medium emitted VOCs (Fig. 3). PTR-ToF-MS is a very sensitive technique, thus it is not surprising that in all sample groups, several VOCs were detected in low concentrations. While all sample groups, including the PDA, produced a number of VOCs in high concentrations, the concentrations of some VOCs were lower in the cultures compared to PDA, thereby indicating VOC consumption. The consumption/production of VOCs strongly depended on Mortierellaceae strains (Fig. 4). There were several VOCs that were produced by some Mortierellaceae strains and consumed by others. The fungal strains frequently consumed and produced VOCs. *P. helmanticensis,* in contrast, rarely consumed, but produced a variety of VOCs. Using generalized linear modelling, we estimated the chance for VOC consumption over VOC production for the different strains and *P. helmanticensis* in pure culture. As the high number of low concentration VOCs might inflate the model, we applied different detection limits and specified several models. All models had a good fit (SI Table S4). Independent of threshold, the binomial model supports that all fungal species and strains were more likely to consume VOCs compared to *P. helmanticensis,* and that the bacterium was a better producer than any fungal species/strain (SI Figs. S7, S8). Compared to the mean concentration across all fungal strains, VOC concentrations were higher in *P. helmanticensis* cultures (p_pairedTTest_ << 0.001). The co-plating of *P.* *helmanticensis* and Mortierellaceae strains did not result in additional VOCs detected compared to all pure cultures (Fig. 4).

Without setting any threshold, only three VOCs (ms105.0299, ms121.0684, ms123.9460; SI Table S5) were consumed by *P. helmanticensis.* Two of them were produced by at least one Mortierellaceae strain in pure culture (Fig. 4), although only in low amounts. For a number of 50 VOCs produced by *P. helmanticensis* there was at least one Mortierellaceae strain able to consume it (SI Table S6). Those VOCs consumed by the fungi could be potential candidates for fungal-bacterial communication. We will address this aspect in subchapter “The volatilome and growth”.

#### The volatilome accurately predicts the taxonomy and cultivation mode

**Figure 5:**
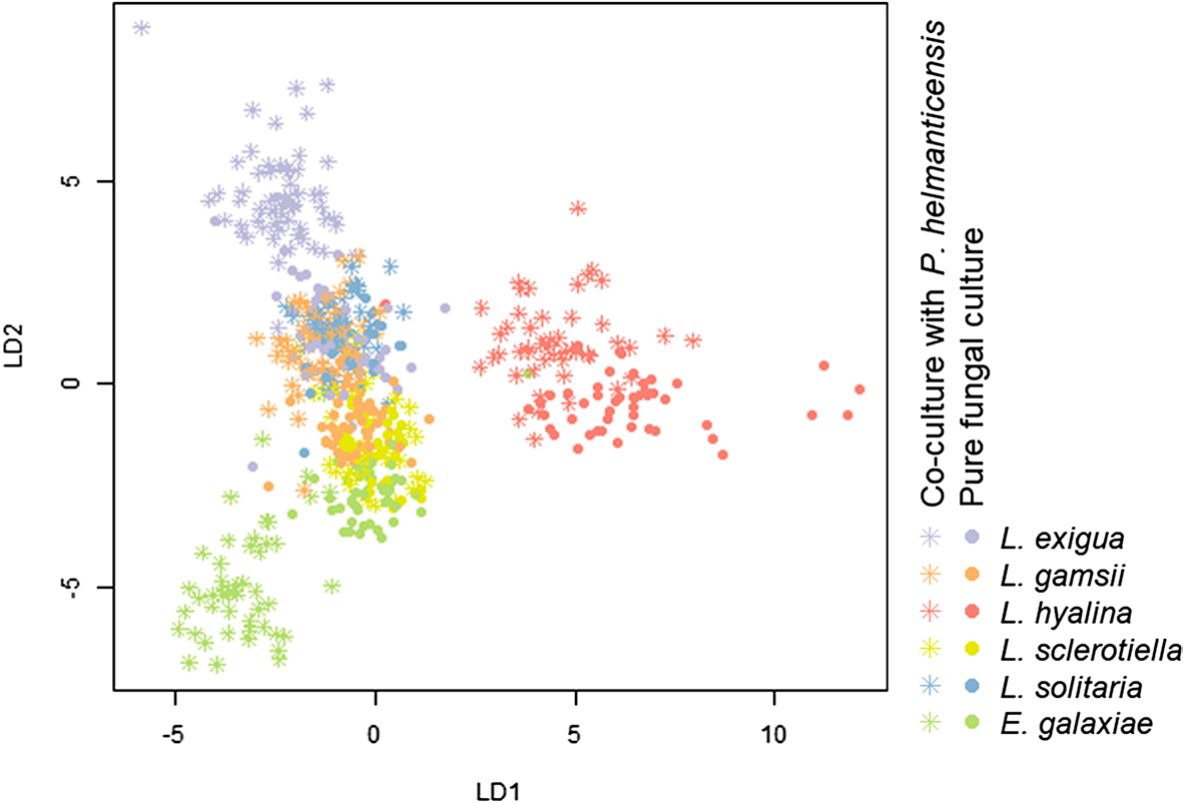
Linear discriminant analysis predictions based on the volatilomes of *Linnemannia* and *Entomortierella* species in fungal pure culture and co-cultivated with *Pseudomonas helmanticensis*. The cultivation mode and taxonomic affiliation to species was predicted. The prediction accuracy was calculated as the number of correctly assigned samples relative to the total size of the test dataset. The accuracy was high: 94% (144/154 samples).

We used linear discriminant analysis (LDA) to predict the taxonomic affiliation (both strain and species) and the cultivation mode from the samples’ volatilomes. For each of the factors, including their combination, the prediction was very accurate (Fig. 5, SI Fig. S9a). The species, including *P. helmanticensis,* could be discriminated with an accuracy of 96% (185/193 samples in the test dataset). The strain was predicted with an accuracy of 84% (156/185 samples in the test dataset). For 94% (144/154) and 61% (103/170) of the test dataset samples, the LDA accurately predicted whether or not the species and strain were co-cultivated with *P. helmanticensis*, respectively.

We expected that in the LDA ordination, those strains with similar changes in growth behaviour following co-plating with *P. helmanticensis* would form a cluster or, along the LDs, shift (or not shift) in the same direction relative to their pure cultures (SI Fig. S9b–f). While such pattern was partially observed and pure cultures could be separated from co-cultures, especially across several LDs, changes in LD scores following co-cultivation were strain-specific.

### The volatilome and growth

#### Models predicting the volatilome contribution of *P. helmanticensis* and Mortierellaceae strains in co-cultures

We applied general linear modelling of the volatilome data in order to (i) link the growth of both bacteria and fungi to the volatilome and (ii) identify individual VOCs that are potentially regulated in the interaction of fungi and *P. helmanticensis*. Using general linear modelling, we implicitly assumed that at least the concentrations of the majority of those VOCs—not every single VOC—were (i) linearly dependent on the cell density and were (ii) not regulated by the bacterial-fungal-interaction. We think this assumption can be hold, because it is unlikely that all VOCs produced/consumed are (i) regulated and (ii) regulated in this particular situation. Considering strain heterogeneity, models were calculated separately for each strain.

Such approach has advantages: (i) the correlation coefficients of the equations are an estimator for growth; and (ii) those VOCs not predicted well by these models violate the assumption of linearity and, therefore, might be regulated.

From our perspective, the most straightforward model would predict the co-culture volatilome from both pure culture volatilomes. However, this model has two disadvantages: First, it might be inaccurate for the bacterial coefficient as the fungal consumption might underestimate the bacterial production. Second, for some fungal strains their coefficients returned insignificant (Table 2), meaning that according to the model, the fungus did not contribute to the co-culture volatilome. As the fungus was visibly growing in all cultures (see also subchapter 3.1), this implies a violation of model assumptions for the models of those strains: if the model is correct, for those strains clearly the entire fungal volatilome was affected by the co-cultivation. Those strains with insignificant coefficients frequently consumed VOCs, but hardly produced VOCs. In other words, numerically their volatilome was characterized by negative concentrations of certain VOCs. *P. helmanticensis,* on the other hand, produced a high number of VOCs in high concentrations (Figs. 3, 4). Due to the presence of *P. helmanticensis* in co-culture, the co-culture VOC concentrations were positive, despite the fungal consumption. Consequently, the fungal contribution to the volatilome was negligible (insignificant coefficient) and the entire volatilome was predicted as a bacterial VOC production.

In order to overcome the disadvantages and weaknesses of this first model (model 1), we calculated two additional models. We compared these additional models to the first model and together all three models provide a conclusive representation of the co-plating experiment (Table 2). Model 2: For each combination of Mortierellaceae strain and *P. helmanticensis*, there was a number of VOCs that were neither produced nor consumed by the fungal strain in pure culture. Therefore, for each unique combination of Mortierellaceae strain and *P. helmanticensis*, we used those VOCs produced only by the bacterium to estimate the bacterial contribution in the co-culture. Model 3: We used model 2 for predicting the bacterial contribution to the co-culture volatilome. This predicted bacterial part of the co-culture volatilome was subtracted from the measured co-culture volatilome. Then, we used those VOCs produced by the respective fungal strain for predicting the theoretical fungal contribution to the co-culture volatilome from the fungal pure culture volatilome.

In the following subsections, we will present the results of the three models in more detail. As a proof of concept, we expected the bacterial coefficients predicted by both approaches to be correlated, and in line with visual observations and growth behaviour (3.1). A conserved mechanism involving VOCs in a potential interaction between *Linnemannia*/*Entomortierella* and *P. helmanticensis,* can be detected by showing a taxonomically conserved pattern of coefficients and VOCs repeatedly diverging from linear predictions across strains. In any case, the inductive effect of any VOC, conserved pattern, or speculated mechanisms needs to be tested in the lab.

#### Mortierellaceae strains limited the growth of *P. helmanticensis*

The bacterial coefficients of models 1 and 2 were numerically comparable and correlated (r = 0.92, *p* << 0.001; SI Fig. S10b). Thus, the intuitive model 1 predicting the co-culture volatilome from both pure culture volatilomes (*P. helmanticensis* and fungal strain), and the model 2 calculated only based on those VOCs produced by the bacterium and neither consumed nor produced by the fungal strain, supported the same conclusion. In both models 1 and 2, the bacterial coefficient was lower than 1, indicating that compared to the pure *P. helmanticensis* culture, the bacterial VOC production was reduced by the fungus (Table 2).

The bacterial coefficients of models 1 and 2 were higher for *E. galaxiae* than for all *Linnemannia* strains (model 2: mean_*Entomortierella*_ = 0.58, mean_*Linnemannia*_ = 0.20, p_TTest_ = 0.00027; comparable for model 1). Some (bacterial) coefficients from both models 1 and 2 were insignificant. In these co-cultivation experiments, the bacterial pure culture volatilome was an (ecologically and/or statistically) insignificant estimate for the volatilome differences observed between the pure and co-culture (Table 2). In other words, the bacterial contribution to the co-culture volatilome was close or indistinguishable from zero. These results were visually confirmed in culture: In those fungal strains with low coefficients, bacterial growth could neither be observed on the co-culture plate, nor under the microscope while observing a sample of fungal hyphae collected from the co-culture. For strains with very high coefficients, however, the bacterial growth was visible on the co-culture plate as well as under the microscope (data not shown).

The predicted coefficients for the bacterial volatilome contribution in co-culture were highly correlated with the centroid distances (r_Spearman|model 1_ = 0.77, *p* = 1.6e−5; SI Fig. S10d) observed between the pure fungal strains and their respective co-cultures.

#### Fungal VOC consumption exceeds their production

The fungal coefficients of models 1 and 3 were numerically comparable and correlated (r = 0.77, *p* = 1.2e−5, SI Fig. S10c) indicating that the intuitive model 1 predicting the co-culture volatilome from both pure culture volatilomes (*P. helmanticensis* and fungal strain) and the model 3 calculated only based on those VOCs produced by fungi supported the same conclusion. It should be noted that the concentrations of the VOCs produced by the fungal strains was often very low (across all strains median concentrations ranged between 0.064 and 0.338 ppbV). The centroid distances and fungal coefficients of both models 1 and 3 were not correlated (p_Spearman|model 1_ = 0.21, p_Spearman|model 3_ = 0.53, SI Fig. S10e). There was no correlation between the daily fungal growth rates and the predicted fungal coefficients (p_Spearman|model 1_ = 0.16, p_Spearman|model 3_ = 0.75).

Comparing the coefficients of models 1 and 3, respectively, the fungal coefficients were lower for *E. galaxiae* than for the *Linnemannia* strains (model 2: mean_*Entomortierella*_ = − 0.11, mean_*Linnemannia*_ = 0.49, p_TTest_ = 0.039; comparable for model 1). There were no differences among *Linnemannia* species.

#### Potentially regulated VOCs

For all three models 1–3 we plotted the predicted co-culture concentrations of VOCs against the measured ones (for an example, see SI Fig. S11). While VOC concentrations usually followed linearity and confirmed model fit, some VOCs were frequently identified as outliers. This is in line with our approach: The residual diagnostics confirmed our ecological model assumptions that the majority of VOCs would be linearly dependent, probably on cell density, and would not be regulated, and that the production of some VOCs in pure culture would differ from their production in co-culture, i.e. they might be regulated.

**Figure 6:**
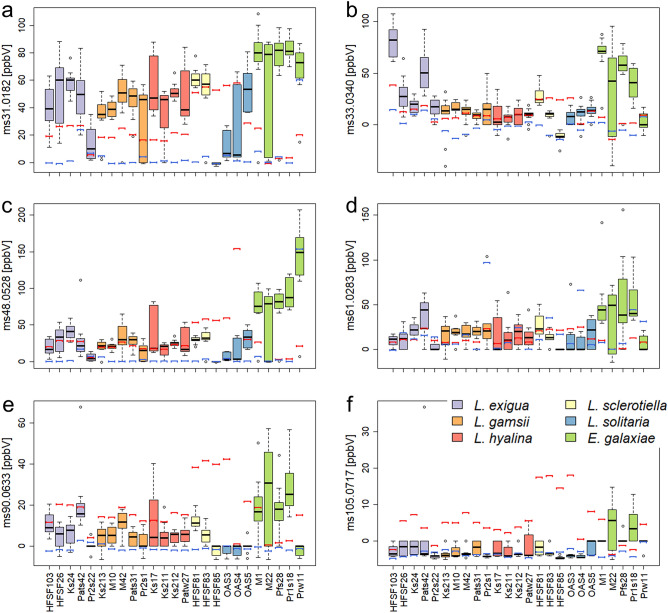
Concentrations of volatile organic compounds (VOCs) that might be potentially regulated in co-cultures of Mortierellaceae strains and *P. helmanticensis*. Those VOCs whose concentrations in co-culture were not predictable from both pure cultures were identified by linear models. The figure visualizes the measured co-culture concentrations in ppbV in all co-culture sample groups. The red bar indicates the predicted VOC concentration from linear modelling (model 1). The blue bar indicates the pure culture concentration. (**a**) ms31.0182 = tentatively annotated (t.a.) as formaldehyde, (**b**) ms33.0340 = t.a. methanol, (**c**) ms48.0528 = ethanol, (**d**) ms61.0283 = acetic acid, (**e**) ms90.0633 = acetoin, (**f**) ms105.0717 = phenylethanol.

Across all models, 14 VOCs did not follow the models’ predictions (SI Table S7). Among these, six VOCs were frequently detected as outliers (7–21 out of 25); their concentrations were often species specific (Fig. 6). Across all species, the VOC tentatively annotated as formaldehyde (ms31.0182, [M + H]^+^) was predicted lower in concentration than it had been measured (Fig. 6a). The VOC (ms33.0340) tentatively annotated as methanol ([M + H]^+^) was predicted lower in concentration than it had been measured in *L. exigua* and *E. galaxiae* (Fig. 6b). For *L. hyalina*, the prediction was accurate. For the other species, the prediction was either too high or too low, depending on the strain. For *L. sclerotiella* and *L. solitaria*, both ethanol (ms48.0528) and acetic acid (ms61.0283, [M + H]^+^) were lower in concentration than predicted by the model, while the measured concentration exceeded the prediction for *E. galaxiae* (Fig. 6c, d, SI Fig. S12 a, b). The VOCs annotated as acetoin (ms90.0633) and phenylethanol (ms105.0717, [M−H_2_O + H]^+^) were predicted higher in concentration than measured for all *Linnemannia* species. In contrast, for *E. galaxiae* the predicted concentration was usually lower compared to the measured concentration (Fig. 6e, f, SI Fig. S12c–f).

## Discussion

Here, we investigated the fungal growth behaviour and volatilomes of 25 strains belonging to *Linnemannia* spp. and *Entomortierella galaxiae* with and without co-plating *P. helmanticensis*. Our results support our hypothesis that fungal growth behaviour and VOC production were taxonomically conserved (Figs. 2, 5), as also observed for other fungal species^14,15^. However, strain heterogeneity was generally large. Co-cultivation of *P. helmanticensis* influenced the growth behaviour, even under standardized conditions not resembling soil conditions. Generally, the bacterium produced VOCs, while the fungus not only produced but also consumed VOCs, including those produced by the bacterium (Figs. 3, 4, SI Figs. S7, S8). This strengthens the hypothesis that VOCs are a means of communication between bacteria and fungi in general, and *P. helmanticensis* and Mortierellaceae in specific. These findings underline the importance of these widespread associations between Mortierellaceae and soil bacteria^18,19^.

The interaction of Mortierellaceae strains and *P. helmanticensis* involves changes in growth behaviour, and very likely VOCs. The centroid distances reflect both, proportionate change of VOC concentrations depending on growth and abundance change of individual VOCs (Fig. 5). The coefficients of the models predicting co-culture volatilomes from the pure culture ones cannot account for the latter result (Table 2). The fungal coefficients were not correlated to the LDA centroid distances (Fig. 5, Table 2, SI Fig. S12). This is intuitive as the difference between the fungal pure culture and its co-culture is not the fungus but the presence of *P. helmanticensis.* The bacterial coefficients, in contrast, were correlated to the centroid distances. This supports the model assumption that the volatilomes depended mainly on the bacterial and fungal biomass. Given that despite the bacterium caused changes in fungal daily radial growth rate and colony morphology, there was neither a correlation between the fungal daily growth rates and the fungal model coefficients nor between the fungal daily growth rates and the LDA centroid distances (SI Fig. S12). Thus, we conclude that the fungal growth did not systematically determine the co-culture volatilome. This does not exclude the opportunity of morphological changes coming with changes in the fungal volatilome or different fungal cells having different VOC expressions. This aspect needs further studying. Nevertheless, even if this is the case, we would still expect a relatively small size effect as those changes were relatively minor. In other words, we conclude that if the fungal VOC production depended on fungal growth or co-culture regulation, this effect was strain specific. Potentially, the fungal volatilome is more versatile among strains than it is generally influenced by the bacterium. The volatilome contribution of *P. helmanticensis,* however, was related to the co-culture volatilome. On the one hand, this supports that the bacterial biomass determined its contribution to the co-culture volatilome. On the other hand, this can support that the fungal strains consumed the bacterial VOCs in a similar manner. Although the fungi also consumed VOCs (Figs. 3, 4), the latter cannot be the sole cause, as the bacterial coefficient of model 2 only included those VOCs neither consumed nor produced by the fungal strain (Table 2). Therefore, we interpret the bacterial coefficients as estimates for bacterial growth, which is in line with the visual observation of the plates, on which no bacterial biomass was observed in those co-cultures in which the bacterial coefficient was insignificant or zero. The additional fact that the bacterial coefficients were not correlated to the fungal daily growth rates suggest that (i) the fungi usually do not directly benefit from or are not directly hampered by the presence of *P. helmanticensis* and (ii) the fungal strains might have controlled the growth of the bacterium.

Our result, that the Mortierellaceae strains did not directly benefit from the presence of *P. helmanticensis,* supports the general assumption that fungi usually counteract the bacterial antagonism by altering their gene expression or even kill them, e.g. by producing antibiotics^33^. The generally low bacterial coefficients, which were comparable to zero in some co-cultures (Table 2), underline a competitive aspect of the interaction among fungi and bacteria. VOCs are also known as potential defence mechanism against pathogens^34,35^. Both, fungi and bacteria, can sensitively react to VOCs produced by other organisms in the environment in terms of growth, spore germination (fungistatis), and bacterial reproduction rate (bacteriostatis)^36,37^. Ethanol, for example, was produced across all species, *P. helmanticensis* and fungal strains, in this study (Fig. 6c). Ethanol production might be a wide-spread evolutionary trait of defence: *Saccharomyces cerevisiae*, e.g. produces higher amounts of ethanol to inhibit *Guignardia citricarpa,* a phytopathogen causing black spot in citrus^38^. Formaldehyde produced in the fruiting bodies of *Mycena rosea* represents an efficient defence mechanism against the fungal mycoparasite *Spinellus fusiger*^39^. *Pseudomonas helmanticensis* produced VOC ms31.0182, tentatively annotated as formaldehyde, in high concentrations (91 ppbV) (Fig. 6a), and in almost all co-cultures the measured concentration of this VOC was much higher than predicted by the models. Interestingly, in those strains, with the lowest bacterial coefficients, formaldehyde was often the highest outlier. Therefore, we speculate that formaldehyde was produced for defence during interactions of some Mortierellaceae and *P. helmanticensis*.

We are aware that both VOC production and pattern will be different under natural conditions. However, we think that the patterns derived from the comparison of pure and co-cultures might provide valuable insights, as in nature, the interaction of Mortierellaceae strains and *P. helmanticensis* cannot be assumed to follow different mechanisms as in vitro. We are also aware that the VOCs not following linearity in the model and therefore identified as potentially regulated, need further experimental confirmation. It is possible, that these were regulated by cell density (following sole fungal and bacterial regulation, respectively) or that these were not directly regulated by the interaction but via more complex mechanisms.

Although *P. helmanticensis* growth was usually reduced by the Mortierellaceae strain, bacterial mobility and dissemination very likely benefit from increased fungal spread. The daily radial growth rate of fungal strains was either unaffected by co-plating (11/27), lower (8/27), or higher (8/27) (Fig. 2, SI Table S2). *L. solitaria* and *E. galaxiae* produced higher amounts of aerial mycelium in co-culture compared to pure culture under nutrient limitation (Fig. 1, SI Fig. 3). This suggests that for some fungal strains the bacterium might have triggered a faster three dimensional spread. However, growth behaviour is generally difficult to evaluate, therefore, it is often impossible to observe whether co-cultivation results in benefit or burden for either partner. The hyphal networks formed by the fungi might provide beneficial hitchhiking options^40^ for *P. helmanticensis* and other bacteria, as they are usually immobile in the absence of a water layer^41^. *Pseudomonas* and also other bacteria such as *Bacillus* species*,* are known to stimulate mycelial growth or fruitbody formation: 1-octen-3-ol and ethylene consuming *P. putida* were found to stimulate the hyphal growth and fruitbody formation in *Agaricus bisporus*^42^. Moreover, ms90.0663, identified as either acetoin or ethyl-acetate, was produced only by *P. helmanticensis,* and it was downregulated in co-culture (Fig. 6e, SI Table S6). Acetoin produced by *P. aeruginosa* induces its motility and decreases biofilm formation^43^. Ethyl-acetate is produced by *Saccharomyces cerevisiae* to inhibit *Botrytis cinerea*^44^, and by *Fusarium oxysporum* while attacking the nematode *Meloidogyne incognita*^45^*.* Thus, this VOC might either (co)regulate bacterial mobility, or serve as defence mechanism.

Acetate was produced in high amounts in both bacterial and fungal cultures, but it was upregulated in co-cultures (Fig. 6d). This supports a metabolic regulation during interaction. Acetate causes acidification of the growth environment, a general defence mechanism. Interacting organisms are thus more resilient than each partner on their own. This further supports our hypothesis that, although not the entire volatilome is determined by the interaction, there might be some general and usually competitive interaction mechanism, including individual VOCs, of fungi and *Pseudomonas spp.*

When also considering that (i) the bacteria might benefit from increased mobility by the fungi despite the likely competitional interaction and that (ii) *P. helmanticensis* produced both a higher number and concentration of VOCs compared to the fungal strains, our results suggest that the bacterium might be more a sender, using VOCs for attraction purposes, than a recipient/interpreter, using fungal VOCs for navigation, while the fungus uses the VOCs for navigation and communication. The methanol production is an example of a VOC supporting this hypothesis (Fig. 6b). Acidotolerant bacteria and fungi are usually regarded as a sink for methanol^46^. However, *P. helmanticensis* produced methanol (m/z 33.0340) in relatively large amounts (mean = 41 ppbV), and also *P. syringae* is producing it as the dominating VOC in pure cultures^47^. Around half of our fungal strains consumed methanol (mean = − 8 ppbV; SI Table S6). This suggests that, at least in the case of *Pseudomonas*, methanol might serve as attractant and carbon source for fungal strains.

Altogether, communication via VOCs appears to be more general and diverse, than specific. This finding explains the promiscuity of *Pseudomonas spp.* when interacting with Mortierellaceae, as detected in our earlier study^19^. For the fungus, communication with a bacterium is very likely more efficient via soluble compounds than via VOCs. Consequently, it might be rewarding to study both the soluble and volatile compounds produced during interaction.

## Supplementary Information

Supplementary Information 1.

Supplementary Information 2.

## Data Availability

All data generated or analysed during this study are included in this published article and its supplementary information files.
