# Supplementary material for: Co-cultivation of Mortierellaceae with Pseudomonas helmanticensis affects both their growth and volatilome
Source: Sci Rep. 2023 Feb 7;13:2213. doi: 10.1038/s41598-023-29134-6 (PMC9905594; doi:10.1038/s41598-023-29134-6)
Supplement: Supplementary file 1 — Supplementary Information 1. [file 41598_2023_29134_MOESM1_ESM.pdf]

Supplementary Information

Co-cultivation of *Linnemannia* with *Pseudomonas helmanticensis* affects both their growth and volatilome

Maraike Probst*[1], Anusha Telagathoti*[1], Bianka Siewert[2], Iuliia Khomenko[3], Emanuela Betta[3], Franco Biasioli[3], and Ursula Peintner[1]

[1]Institute of Microbiology, Universität Innsbruck, Technikerstrasse 25, 6020 Innsbruck, Austria

[2]Institute of Pharmacy, Universität Innsbruck, Center for Chemistry and Biomedicine, Center for Molecular Biosciences Innsbruck (CMBI), Innrain 80 - 82/IV, 6020 Innsbruck, Austria

[3]Fondazione Edmund Mach, Research and Innovation Centre, Via Edmund Mach 1, 38010 San Michele all'Adige, Italy

*these authors contributed equally

Correspondence: Probst.maraike@gmail.com

The entire Supplementary consists of figures, tables, scripts and datasets. This Supplementary file contains figures only. For tables, please check the other parts of the supplement. For scripts and data, please check the Github project (Maraikep/Mortierellaceae_VOCs). Please feel free to contact us if there are any queries.

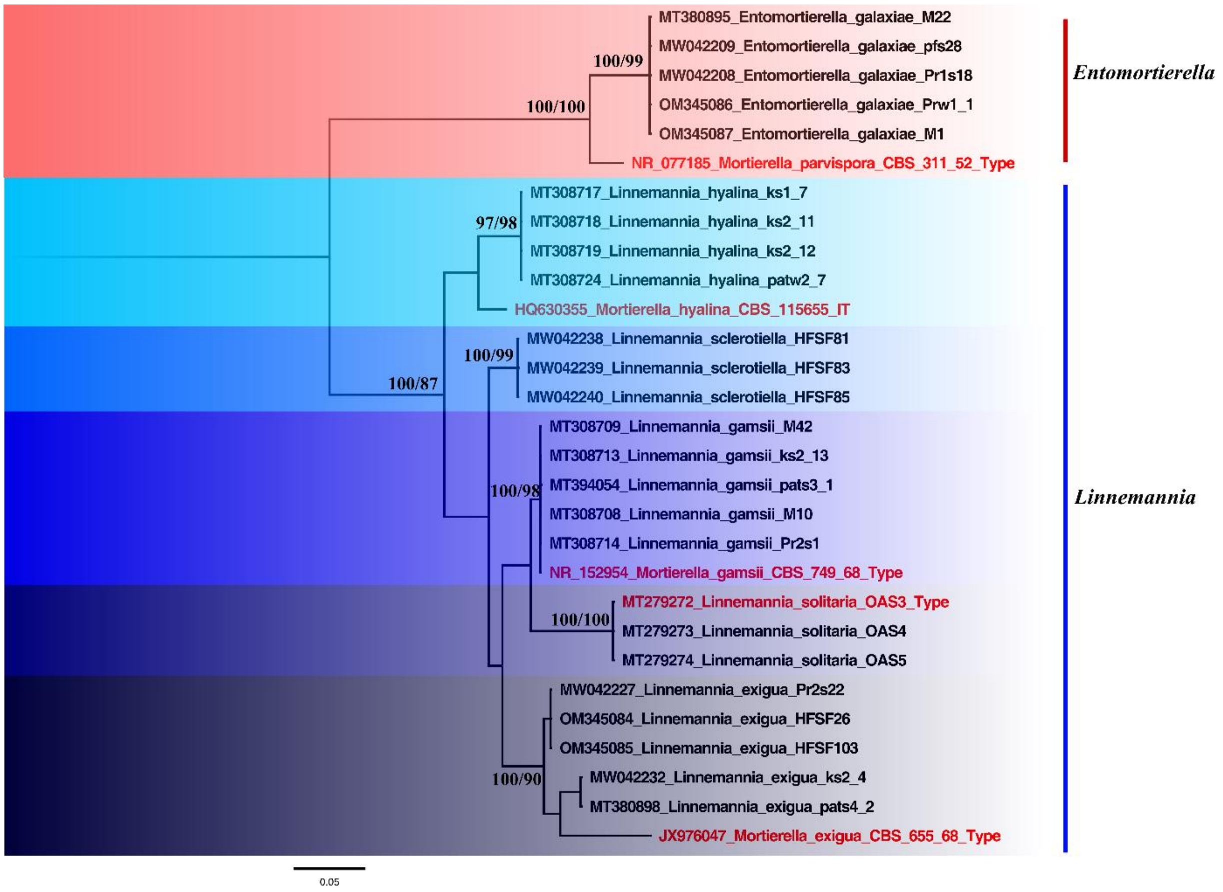

SI Figure 1 Phylogenetic analysis of strains studied here (log likelihood -1962.79) based on rDNA-ITS sequences. For branches close to the origin, branch ≥70 is shown above the branches as Bayesian posterior probabilities/Parsimony-based bootstrap values. Sequences originating from type specimens are highlighted in red.

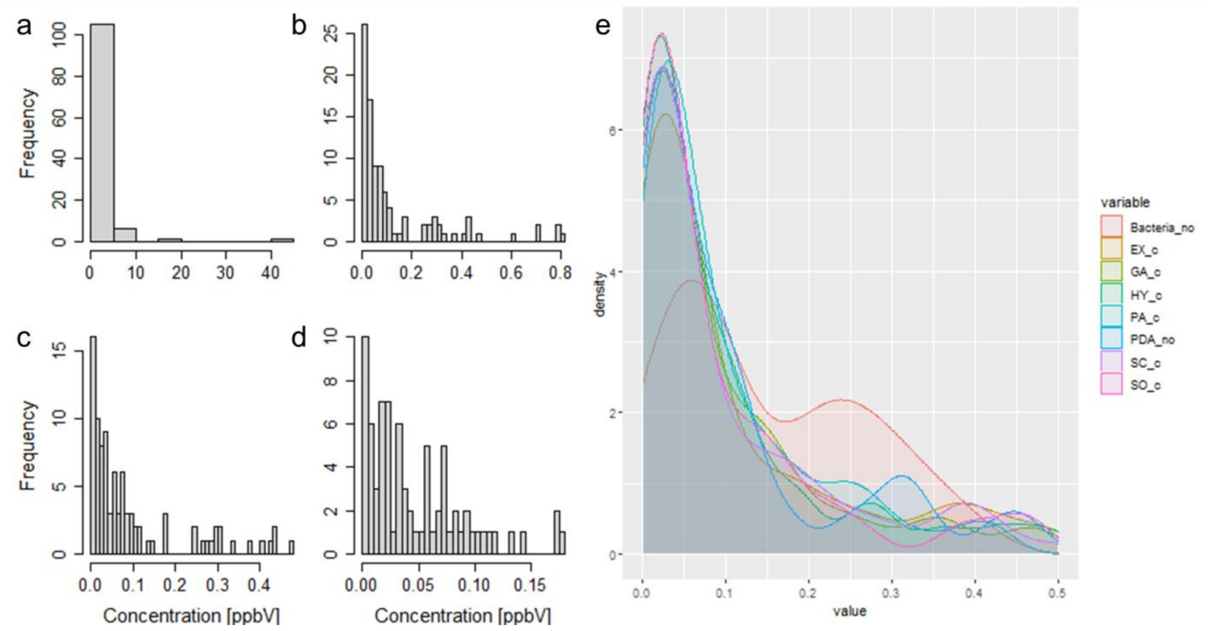

SI Figure 2 Distribution of mass peak concentrations. (a-d) Concentrations of mass peaks detected in PDA medium. (a) All mass peaks are shown. (b) All mass peaks below a concentration of 1 ppbV are shown. (c) All mass peaks below a concentration of 0.5 ppbV are shown. (d) All mass peaks below a concentration of 0.2 ppbV are shown. (e) The density distribution of all mass peak concentrations below 0.5 ppbV are shown for all pure cultures grouped by their respective species.

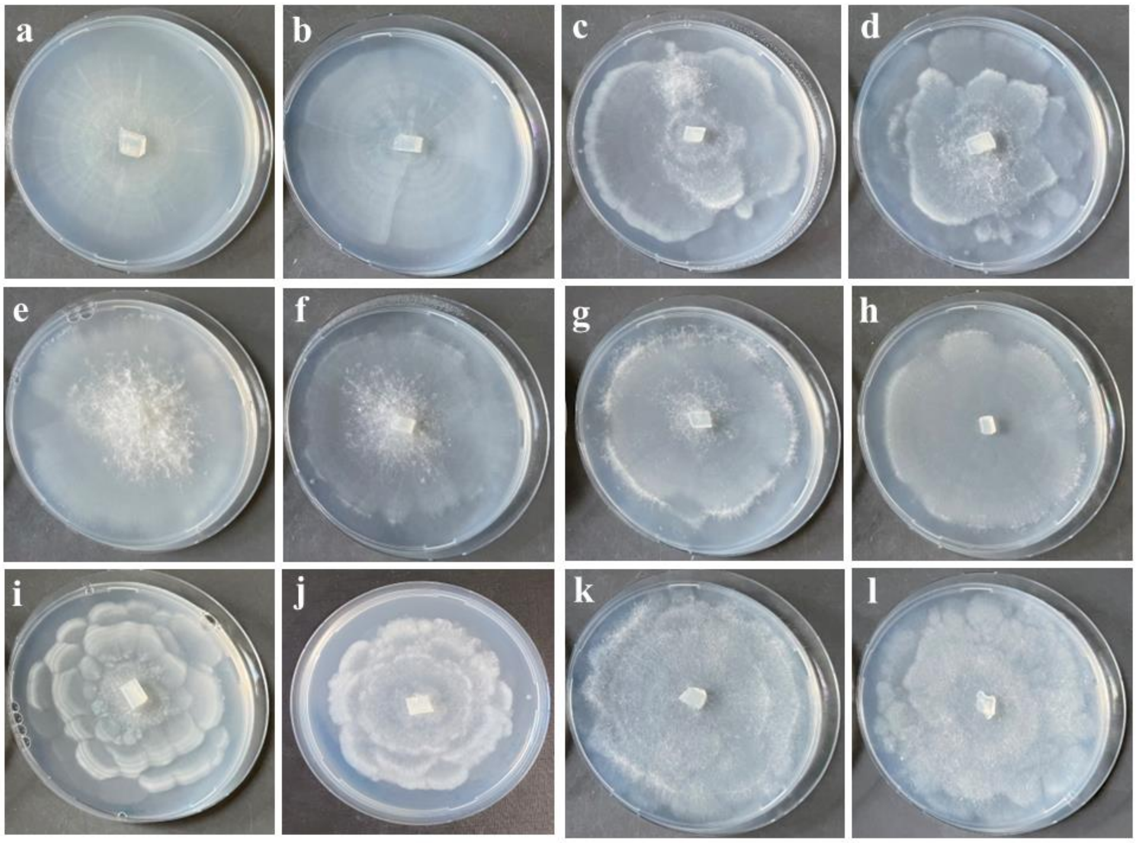

SI Figure 3 Morphology of *Linnemannia* and *Entomortierella* species on LCA in pure (second and forth column) and co-culture with *Pseudomonas helmanticensis* (first and third column) incubated at 10 °C for 10 d. One exemplary strain was randomly selected for each species. (a, b) *E. galaxiae*, (c, d) *L. sclerotiella*, (e, f) *L. gamsii*, (g, h) *L. hyalina*, (i, j) *L. solitaria*, (k, l) *L. exigua*.

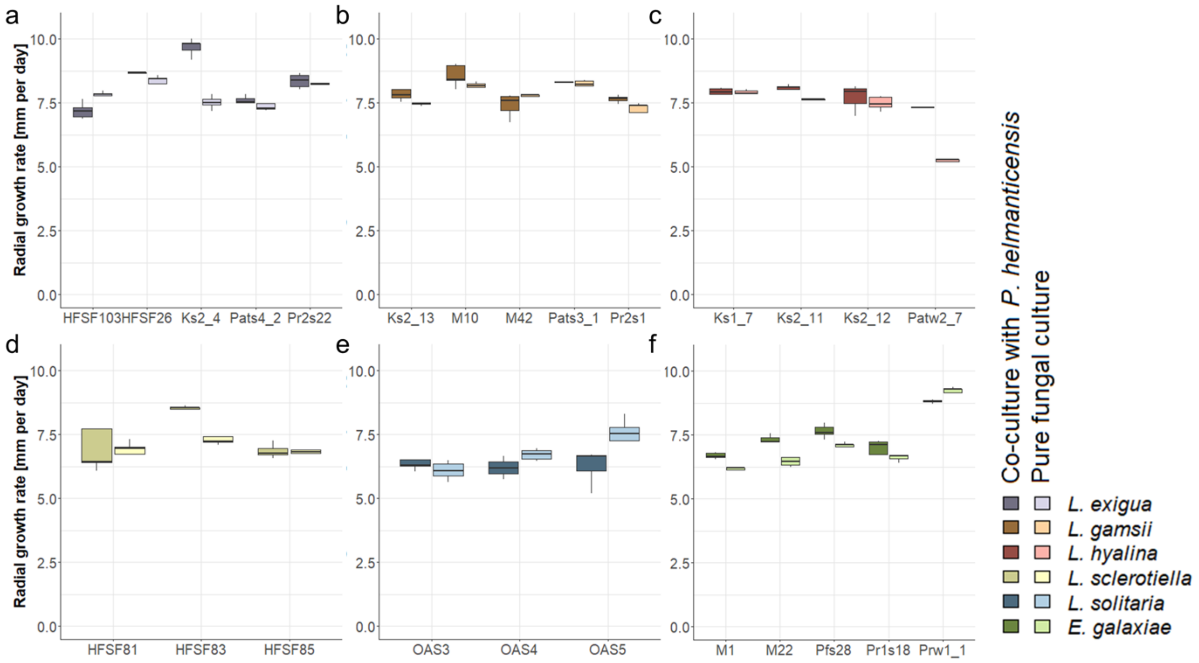

SI Figure 4 Daily radial growth rates of pure *Linnemannia* species (a-e) and *Entomortierella galaxiae* (f) cultures as well as their co-cultures with *Pseudomonas helmanticensis* on nutrient poor LCA medium.

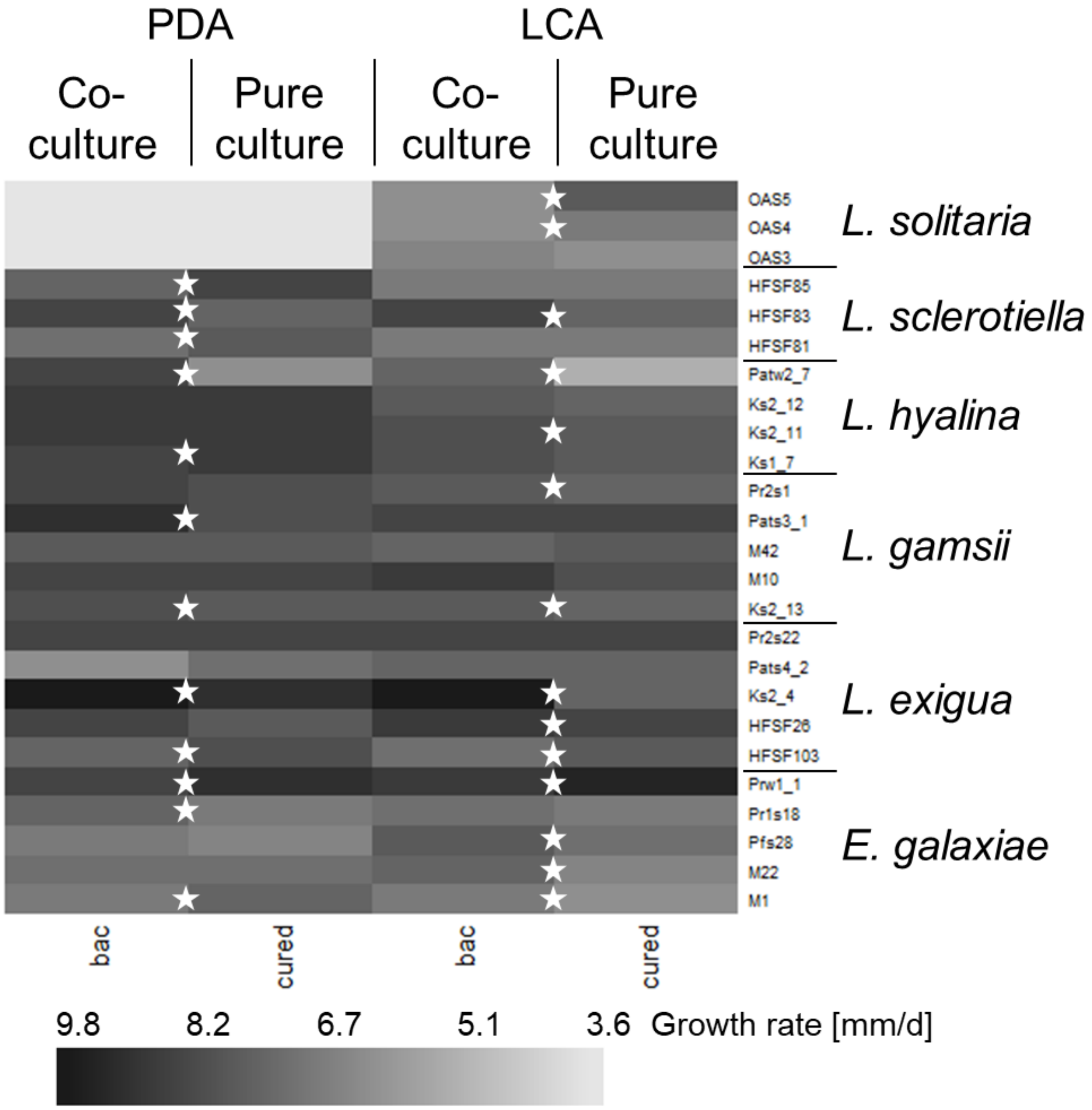

SI Figure 5 Growth of *Linnemannia* and *Entomortierella* strains in pure culture compared to their co-cultures with *P. helmanticensis*. Growth was measured as daily radial growth rate on nutrient rich PDA and poor LCA medium. White stars indicate significant differences among pure and co-cultures ($p < 0.05$).

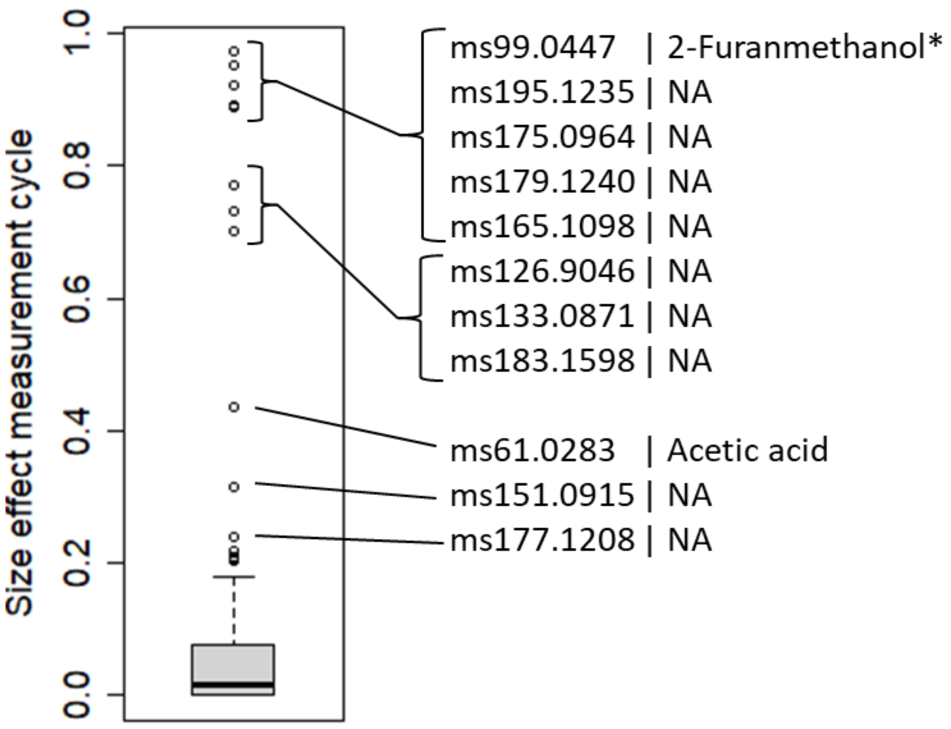

SI Figure 6 Stability of the volatilomes over time. Changes in volatiles concentrations were predicted by all experimental factors (species, strain (nested in species), cultivation mode, and measurement cycle) using analysis of variance. For those volatiles, for which measurement cycle was a significant predictor, the size effect of the factor was calculated using ANOVA sum of squares. The size effect was expressed as percentage of variance explained by the factor measurement cycle and visualised as a boxplot. In conclusion, the factor measurement cycle was a poor predictor, indicating that the volatile production was stable over the time of measurement. NA = no annotation of the mass peak could be tentatively assigned.

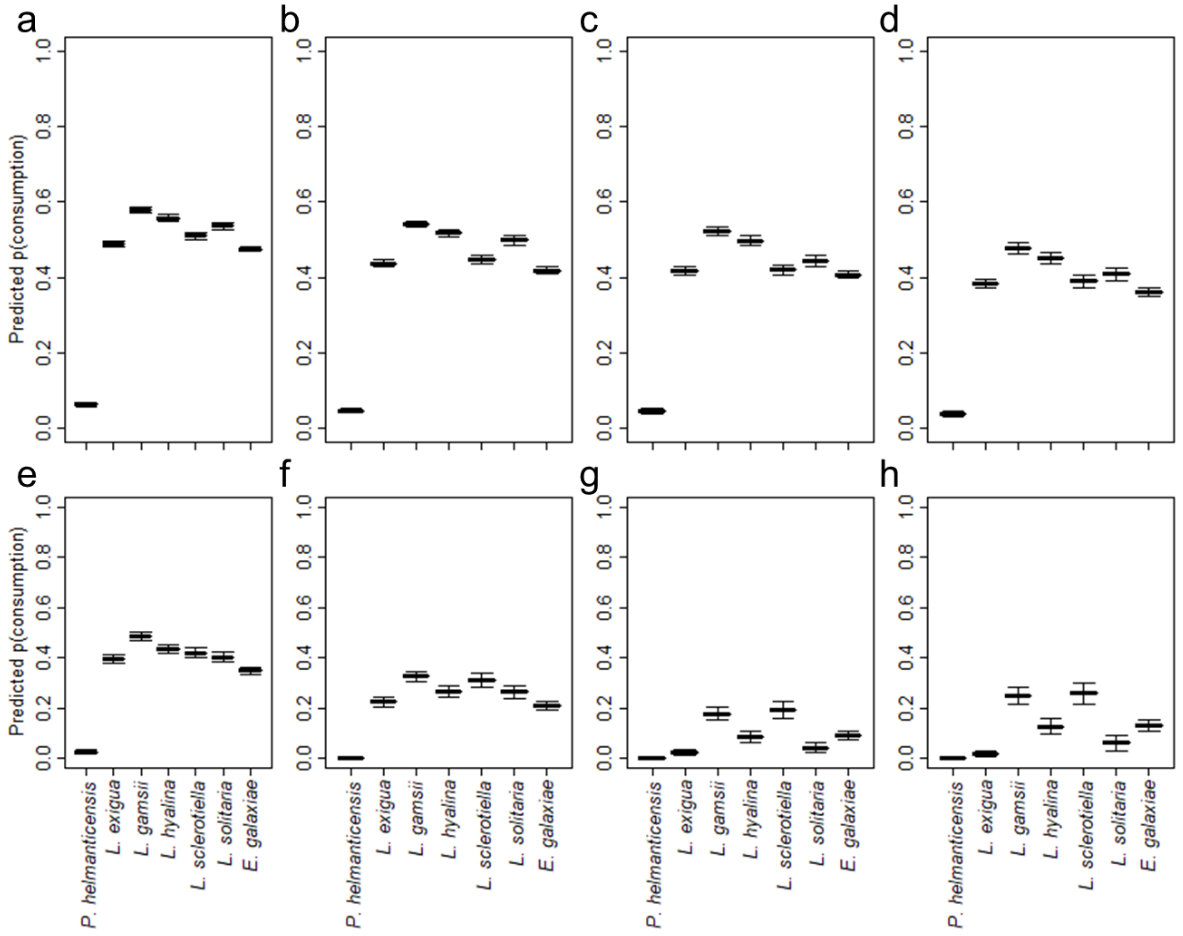

SI Figure 7 Estimated probability of mass peak consumption by species. The probability of mass peak consumption was estimated using generalized linear models assuming binomial distribution and using logit as link function. Species was used as fixed factor in the linear predictor. Different panels visualize different detection limits of volatile organic compounds detected by the PTR-ToF-MS. (a) no threshold set, (b) 0.05 ppbV, (c) 0.2 ppbV, (d) 0.5 ppbV, (e) 1 ppbV, (f) 5 ppbV, (g) 10 ppbV, (h) 20 ppbV. Differences are significant on $p < 0.05$. *L. = Linnemannia; E. = Entomortierella, P. = Pseudomonas.*

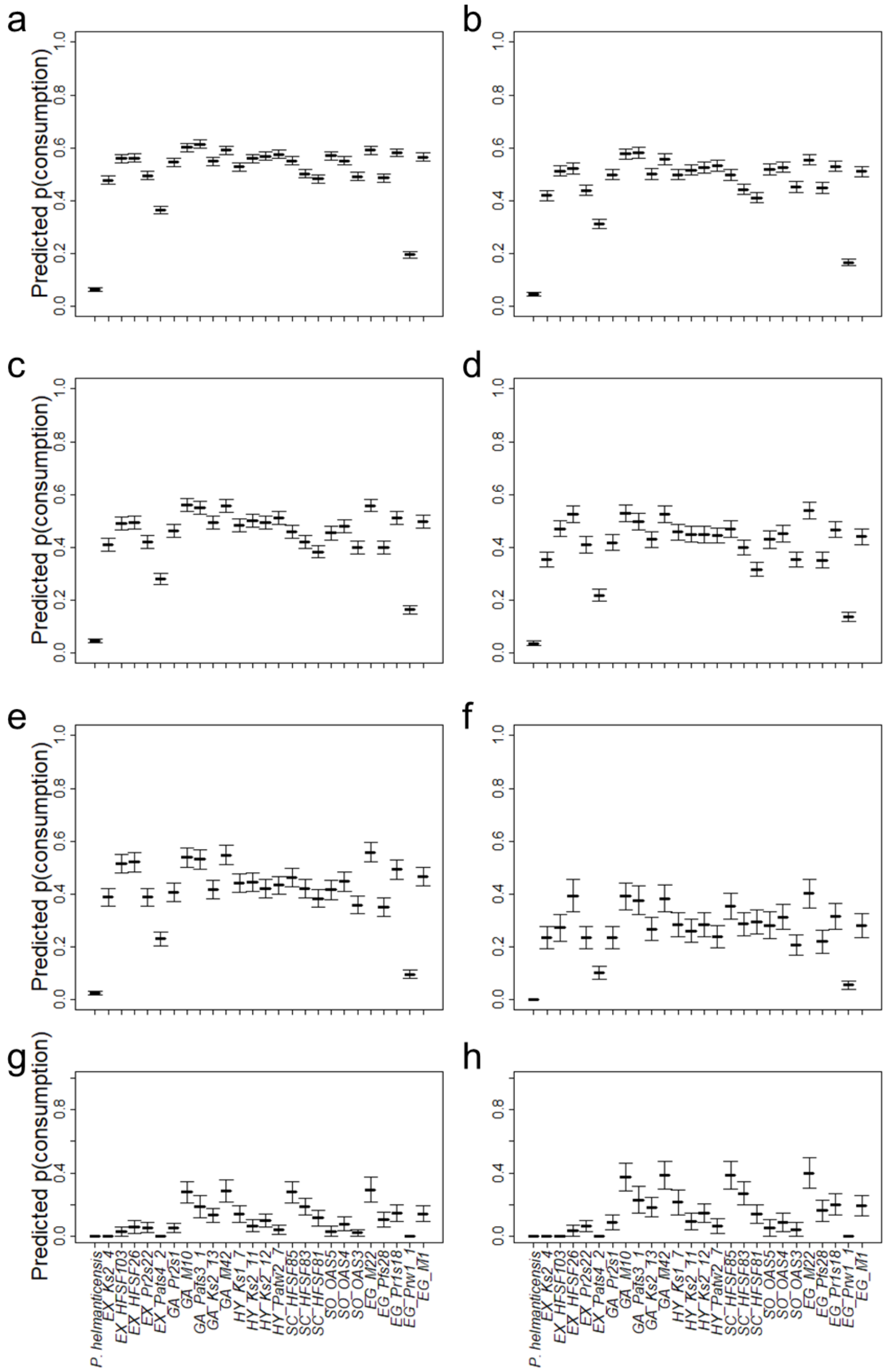

SI Figure 8 Estimated probability of mass peak consumption by strains. The probability of mass peak consumption was estimated using generalized linear models assuming binomial distribution and using logit as link function. Species was used as fixed factor in the linear predictor. Different panels visualize different detection limits of volatile organic compounds detected by the PTR-ToF-MS. (a) no threshold set, (b) 0.05 ppbV, (c) 0.2 ppbV, (d) 0.5 ppbV, (e) 1 ppbV, (f) 5 ppbV, (g) 10 ppbV, (h) 20 ppbV. Differences are significant on $p < 0.05$. Sample groups are ordered by species affiliation. EX = *Linnemannia exigua,* GA = *L. gamsii,* HY = *L. hyalina,* SC = *L. sclerotiella,* SO = *L. solitaria,* EG = *Entomortierella galaxiae*.

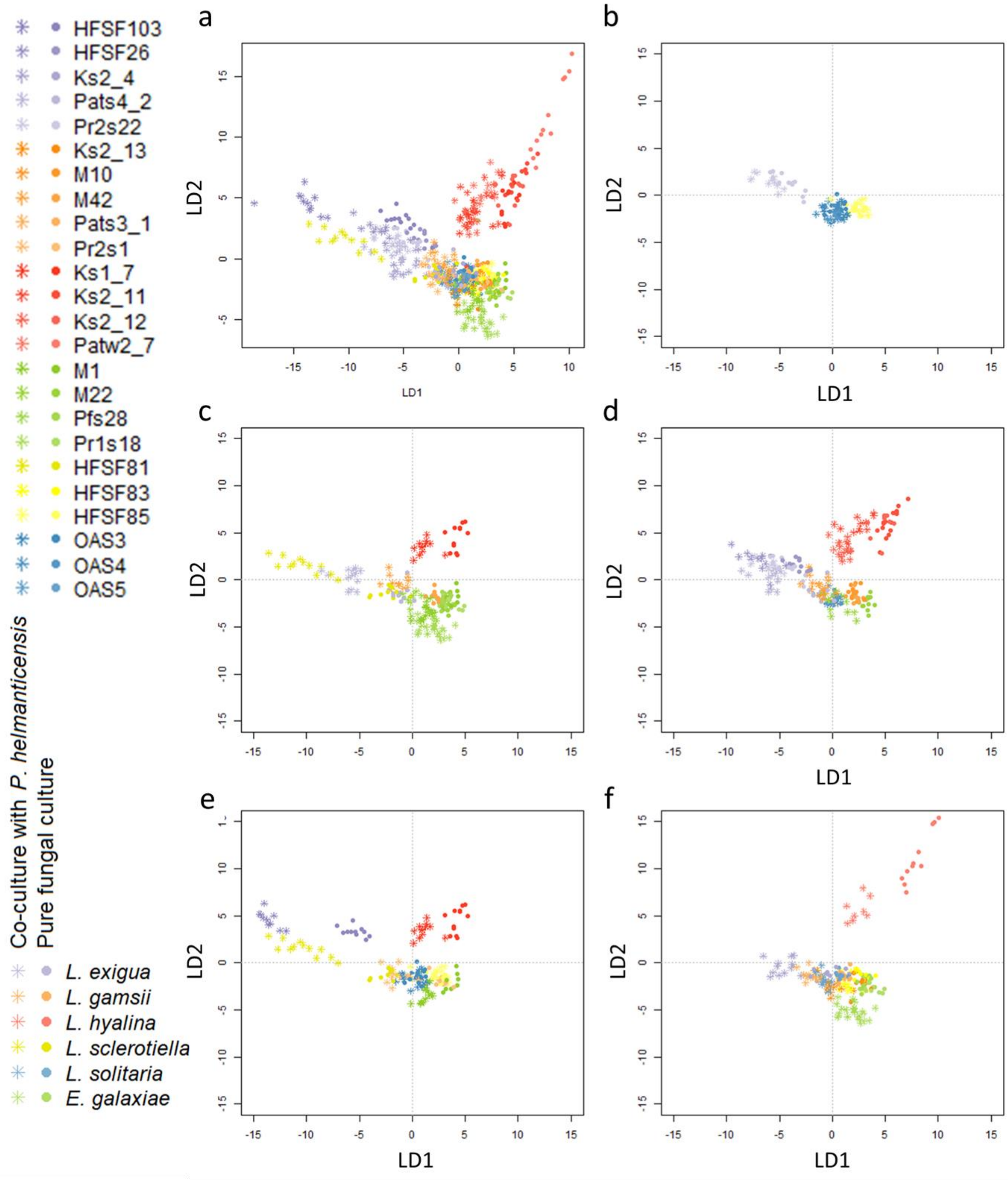

SI Figure 9 Linear Discriminant Analysis (LDA) predicting (a) the strain and cultivation mode (pure and co-cultivation with *P. helmanticensis*, respectively) of the samples; (b) Only strains with a model fit and coefficient close or indistinguishable from zero are shown; (c) Only strains with a good model fit and high coefficient are shown; (d) Only strains that did not show any significant differences in growth behaviour among pure and co-cultivation; (e) Only strains whose daily radial growth rate was lower in co-culture conditions than in pure culture; (f) Only strains whose daily radial growth rate was higher in co-plating conditions than in pure culture. *L. = Linnemannia; E. = Entomortierella.*

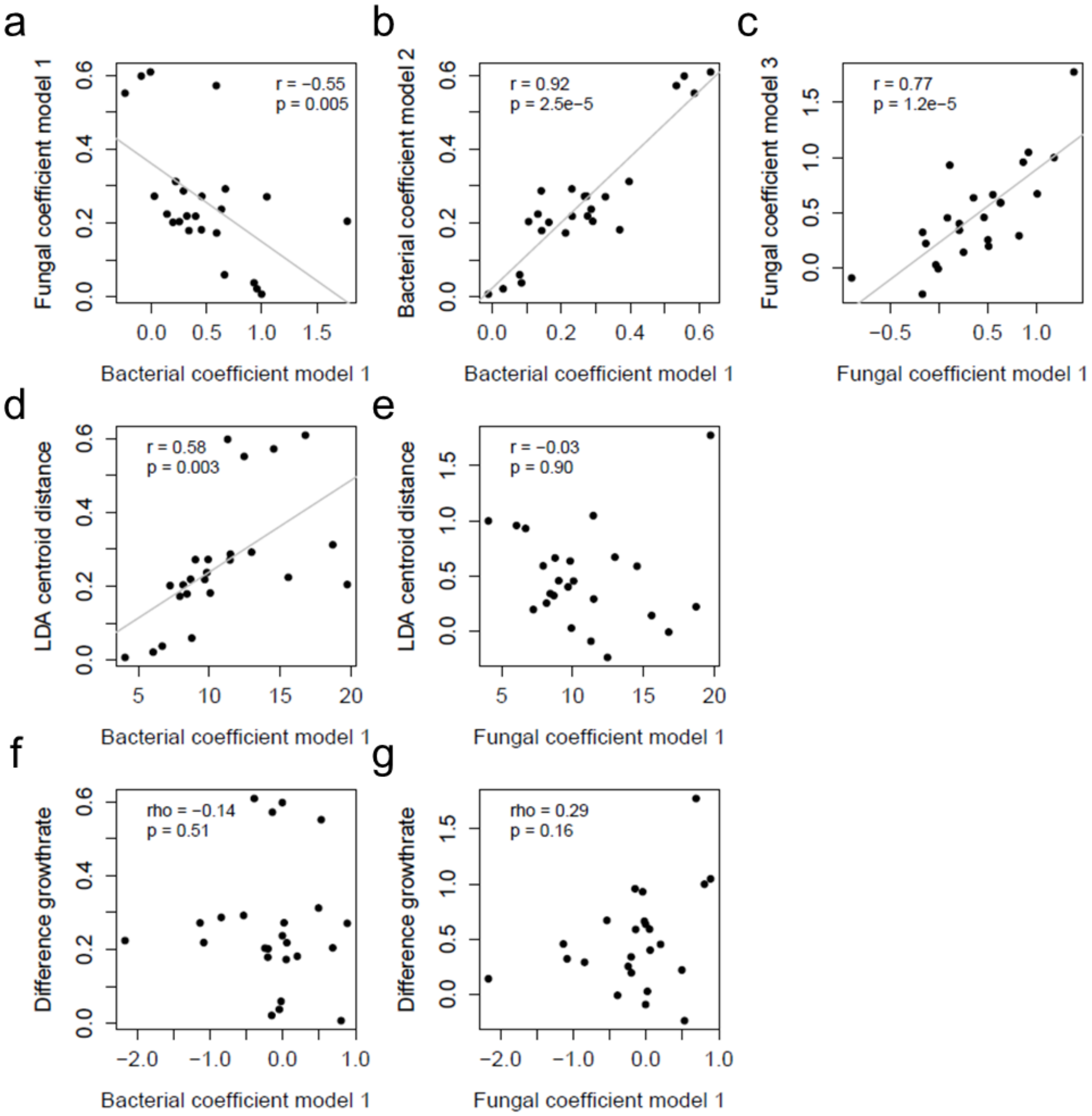

SI Figure 10 Correlation analysis. The main correlation analysis used across aspects of this study. In panels (a-e) Pearson correlation was used as these data can be assumed normally distributed. In panels (f) and (g) Spearman rank correlation was applied as these data are not normally distributed.

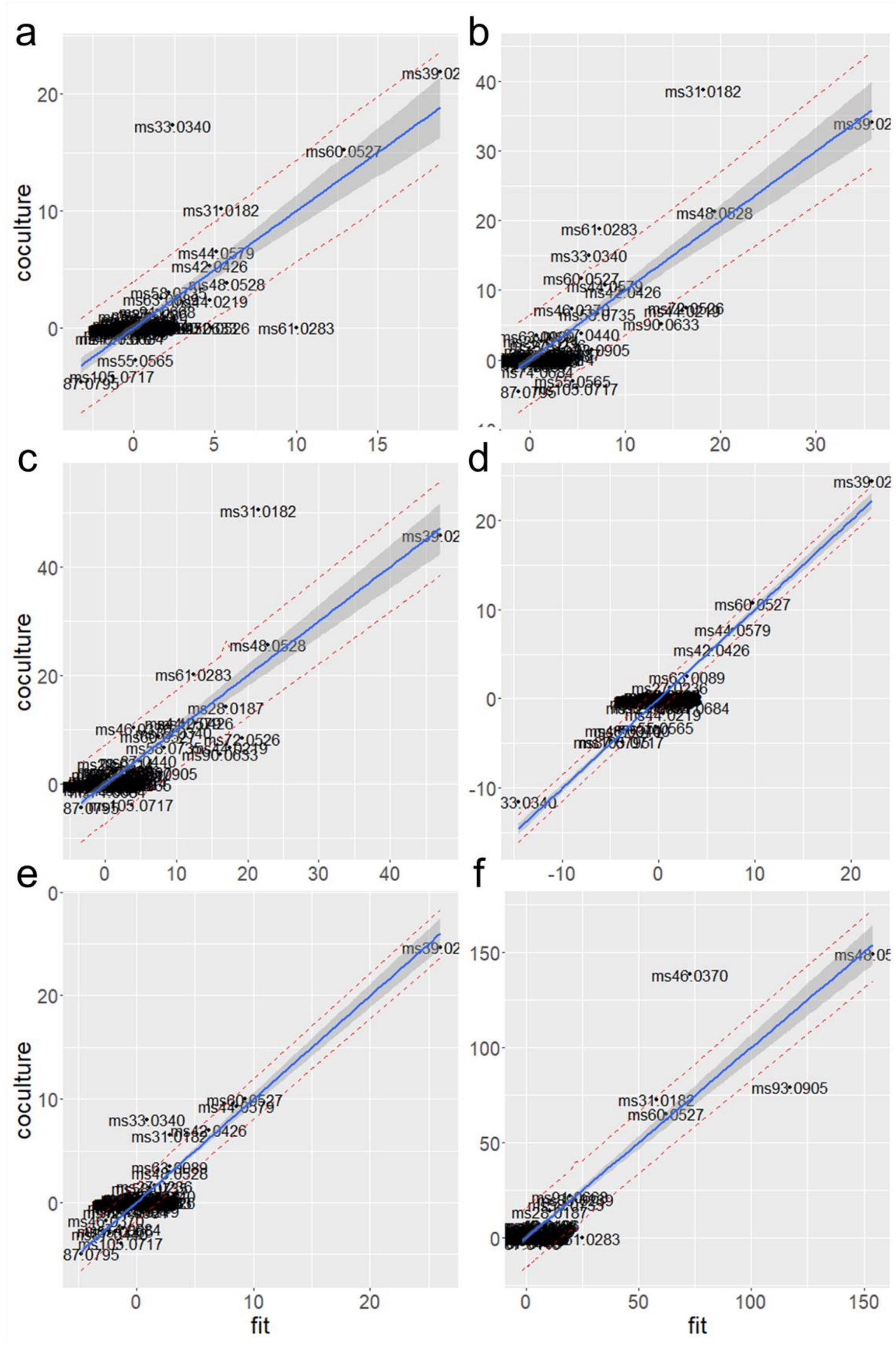

SI Figure 11 Co-culture concentrations of volatile organic compounds predicted by the model versus measured values. Models were calculated individually for each strain. For each species of *Linnemannia* and *Entomortierella* investigated, an exemplary strain was selected for illustration. (a) *L. exigua,* strain Pr2s22, (b) *L. gamsii*, strain M10, (c) *L. hyalina*, strain Ks2_12, (d) *L. sclerotiella*, strain HFSF85, (e) *L. solitaria*, strain OAS3, (f) *E. galaxiae,* strain Prw1_1.

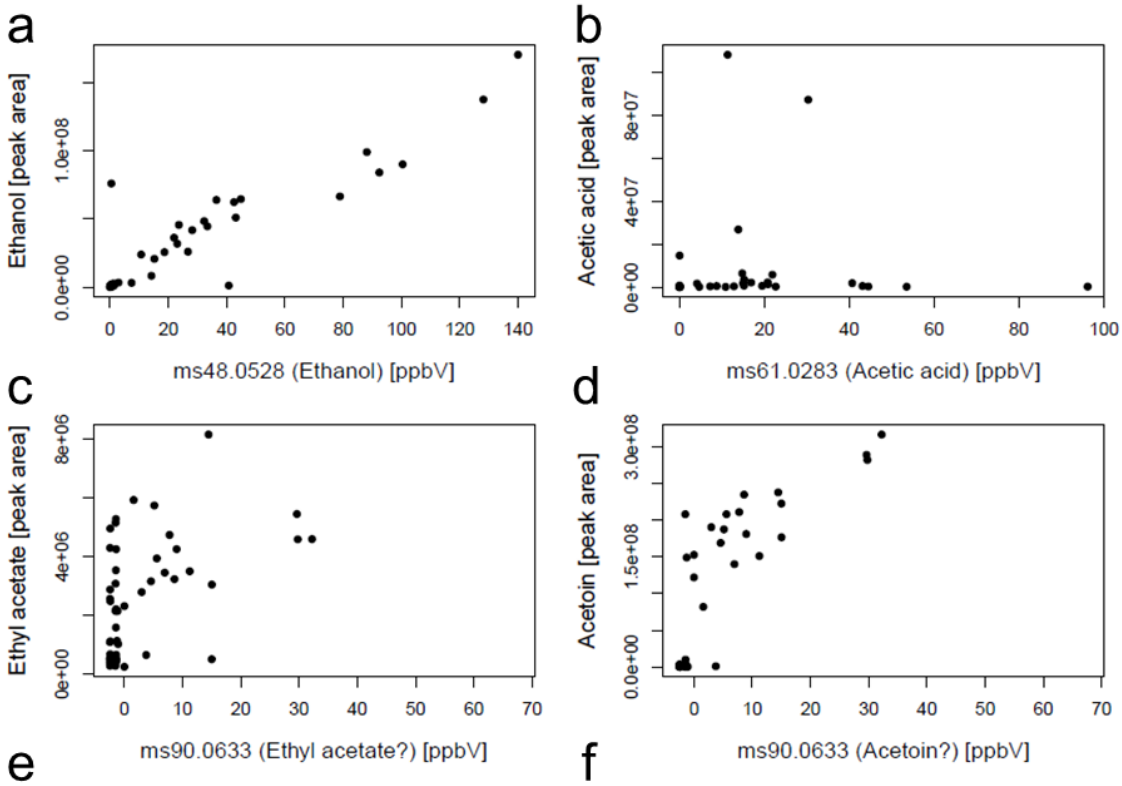

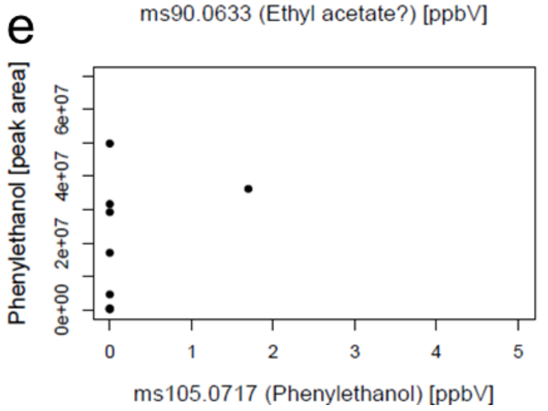

f

	Annotation/ Identification	Pearson r	p-value	Spearman rho	p-value
**a**	Ethanol	0.928	< 0.001	0.907	< 0.001
**b**	Acetic acid	0.015	0.934	0.391	0.02
**c**	Ethyl acetate?	0.467	< 0.001	0.441	0.002
**d**	Acetoin?	0.821	< 0.001	0.787	< 0.001
**e**	Phenylethanol	0.453	0.001	0.527	< 0.001

SI Figure 12 Correlations of PTR-ToF-MS and GC-MS measured concentrations for selected volatile organic compounds (VOCs). As PTR-ToF-MS measurements only allow for tentative VOC annotations, GC-MS measurements of one representative technical replicate were performed for all sample groups. For PTR-ToF-MS, VOCs were identified according to their masses. For GC-MS, VOCs were identified based on mass spectra matching with the standard NIST/EPA/NIH (NIST 14) and Wiley 7th Mass Spectral Libraries, and linear retention indices (LRI) compared with the literature. For those VOCs that were potentially regulated during co-cultivation of the *Linnemannia* and *Entomortierella* strains with *P. helmanticensis* as identified by linear models, their PTR-ToF-MS concentrations and GC-MS concentrations were correlated based on their tentative annotation and identification, respectively. The identity of the PTR-ToF-MS VOC (a-e) was confirmed if their correlation to GC-MS concentrations was significant and high (f). The figure visualizes the correlations of those VOCs.
